# Spatial transcriptomics of the lacrimal gland features macrophage activity and epithelium metabolism as key alterations during chronic inflammation

**DOI:** 10.3389/fimmu.2022.1011125

**Published:** 2022-10-17

**Authors:** Olivier Mauduit, Vanessa Delcroix, Takeshi Umazume, Cintia S. de Paiva, Darlene A. Dartt, Helen P. Makarenkova

**Affiliations:** ^1^ Department of Molecular Medicine, The Scripps Research Institute, La Jolla, CA, United States; ^2^ The Ocular Surface Center, Department of Ophthalmology, Baylor College of Medicine, Cullen Eye Institute, Houston, TX, United States; ^3^ Schepens Eye Research Institute of Massachusetts Eye and Ear, Harvard Medical School, Boston, MA, United States

**Keywords:** lacrimal gland, chronic inflammation, visium, lipid metabolism, RNA sequencing, TYROBP, macrophages, spatial transcriptomics

## Abstract

The lacrimal gland (LG) is an exocrine gland that produces the watery part of the tear film that lubricates the ocular surface. Chronic inflammation, such as Sjögren’s syndrome (SS), is one of the leading causes of aqueous-deficiency dry eye (ADDE) disease worldwide. In this study we analyzed the chronic inflammation in the LGs of the NOD.B10Sn-H2b/J (*NOD.H-2b*) mice, a mouse model of SS, utilizing bulk RNAseq and Visium spatial gene expression. With Seurat we performed unsupervised clustering and analyzed the spatial cell distribution and gene expression changes in all cell clusters within the LG sections. Moreover, for the first time, we analyzed and validated specific pathways defined by bulk RNAseq using Visium technology to determine activation of these pathways within the LG sections. This analysis suggests that altered metabolism and the hallmarks of inflammatory responses from both epithelial and immune cells drive inflammation. The most significant pathway enriched in upregulated DEGs was the “TYROBP Causal Network”, that has not been described previously in SS. We also noted a significant decrease in lipid metabolism in the LG of the *NOD.H-2b* mice. Our data suggests that modulation of these pathways can provide a therapeutic strategy to treat ADDE.

## Introduction

Sjögren’s syndrome (SS) is a systemic autoimmune disease that affects the entire body but is identified by its two most common symptoms — dry eye and dry mouth (1, 2). SS affects 0.5% to 1.0% of the population (3) of any age, but symptoms usually appear between the ages of 41 and 60 (4), and women are more frequently affected (5, 6). Dry eye and dry mouth manifestations can precede the diagnosis of SS by several years (7). SS could be *primary* or *secondary* (8). In contrast to secondary SS, primary SS (pSS) occurs in the absence of another underlying autoimmune disease, such as systemic lupus erythematosus, rheumatoid arthritis, or diabetes.

Low tear volume is a hallmark of dry eye in SS and relates to secretory dysfunction of the lacrimal gland (LG) due to spontaneous development of focal lymphocytic infiltrates adjacent to blood vessels and excretory ducts in the LG (1, 9, 10). LG chronic inflammation is characterized by the infiltration of lymphocytes forming foci, that leads to the loss of LG secretory cells (11, 12). The foci are predominantly composed of B and T lymphocytes and to a lesser degree other cell types (13, 14). The increased levels of the pro-inflammatory cytokines IL-1β, IL-6, tumor necrosis factor (TNF)-α, and IFN-γ that have been reported in the LG of patients with SS and mouse models of SS (10, 13, 15, 16) are mostly secreted by the infiltrating lymphocytes.

Despite the well-characterized clinical manifestations associated with pSS, the underlying pathogenesis of this disease remains largely unknown (6). Furthermore, pSS treatments are associated with topical lubrication (artificial tears) and anti-inflammatory drugs, but no medication addresses the underlying causes (17, 18).

During the last several decades, several mouse models have been developed to study various aspects of human SS disease (11, 19–21). The nonobese diabetic (*NOD*) mouse remains one of the most extensively characterized and well-studied mouse models to investigate the pathogenesis of SS. However, the development of diabetes prior to autoimmune exocrinopathy in the *NOD/LtJ* mouse suggests that it is an excellent model of secondary, but not primary, SS complications (21, 22). By contrast, the diabetes-resistant *NOD.H-2b* mouse strain, in which the I-Ag7 segment of the MHC region has been replaced by the H2b haplotype of *C57BL/10SnJ* mice, does not have autoimmune diabetes, but displays severe lymphocytic infiltration and dysfunction of the lacrimal and salivary glands (23, 24). The *NOD.H-2b* mouse is a primary SS mouse model, that similar to humans, retains the characteristic features of lymphocytic infiltration and dysfunction of the LG. These mice show reduced lacrimal and salivary gland secretion, formation of mononuclear lymphocytic infiltrations, and the presence of the SSA and SSB antibodies (11, 24). A recent study reported scRNA-seq analysis of the salivary submandibular glands (SMG) of the *NOD.H-2b* (25). This study identified inflammation-induced changes in multiple cell populations of the SMG, suggesting the complex nature of the cellular responses to chronic inflammation. Although some of the histological and physiological features of LG disease in these mice have been described (2, 26), no extensive studies of the LG transcriptome in these mice have been performed.

In our study we used the *NOD.H-2b* male mice to characterize changes in the LG due to disease progression. Although the *NOD.H-2b* mice develop the disease in both sexes, the disease in males is more consistent and robust, and is detected in LGs as early as 1.5-2.0 months (M) of age, while females develop disease much later around 4-6M of age. We characterized the transcriptome of LG by bulk RNA sequencing to determine biological pathways altered during disease progression. To better understand the molecular and cellular mechanisms driving this autoimmune disease, we used VISIUM spatial gene expression technology to map gene expression on LG cryosections. We produced the first spatial transcriptomic atlas of LG in BALB/c mice and *NOD.H-2b* mice that allowed us to interpret cellular expression levels and pathways in the context of tissue morphology. Finally, we validated some of our findings using immunostaining.

## Results

### NOD.H-2b males develop inflammation earlier than females

The *NOD.H-2b* mouse strain was described in many publications (27, 28) and displays many features of human SS, including exocrine gland dysfunction and leukocyte infiltration of the salivary glands and LGs. Both *NOD.H-2b* males and females have elevated IgM and IgG autoantibodies in serum and immune foci in exocrine glands at 6 months old, which is considered as the clinical stage (2). However, there is no consistent description of timing and manifestation of the disease in the LG of males compared to females (2, 24, 29).

**Figure 1:**
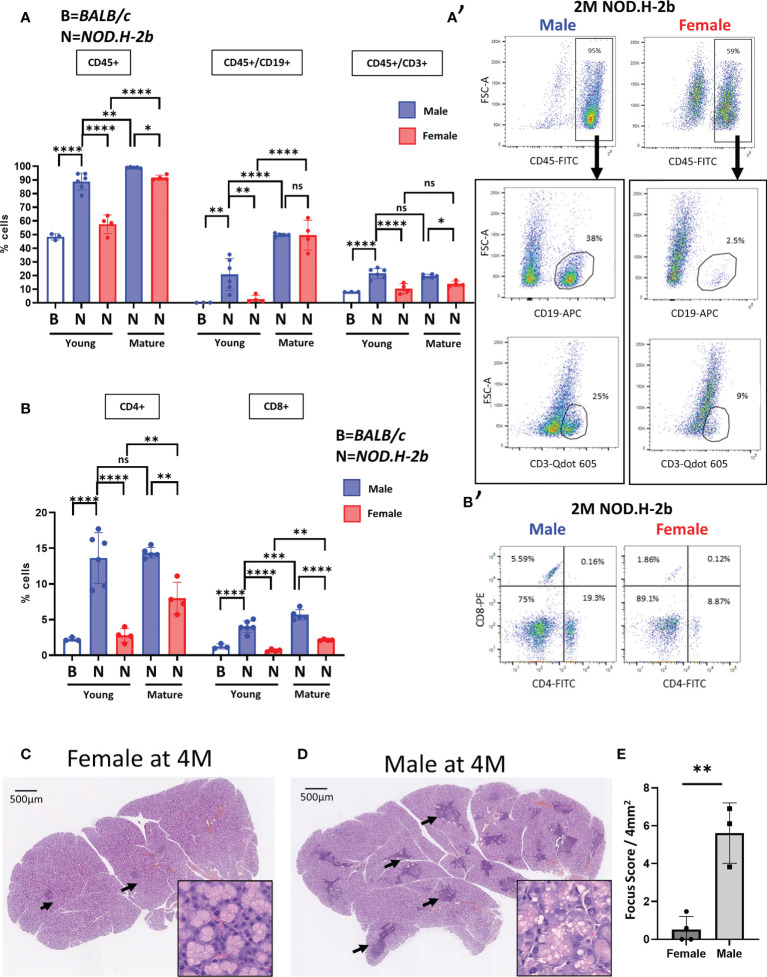
Increase of B and T cells proportion in Male NOD.H-2b mice with age. CD45^+^ immune cells were analyzed by flow cytometry of LGs from BALB/c **(B)** and NOD.H.2b (N) mice. **(A, A’)** Comparison of B cells (CD19^+^) and T cells (CD3^+^) between LGs of NOD.H-2b male and female have been performed at 2 and 6M within immune population (CD45^+^) 2M old BALB/c have been used as control (empty bar). A representative plot at 2M is shown for male and female **(A’)**. **(B)** Histogram showing proportion of CD4^+^ and CD8^+^ cells. A representative plot at 2M is shown for male and female **(B’)**. **(A, B)** Values are shown as mean ± SD. Statistical significance assessed with ANOVA test. Adjusted P-value significance: *: p <0.05; **: p <0.01; ***: p <0.001; ****: p <0.0001; ns: non-significant. **(C, D)** H&E staining of LGs from NOD.H-2b female **(C)** and male **(D)** showed immune infiltrates (black arrows). **(E)** Histogram representing focus score of LGs between male and female at 4 months Plot shows mean ± SD. Statistical significance assessed with an unpaired t-test (**: p=0.0021).

To clarify this issue, we analyzed the LGs of both males and females by flow cytometry. Analysis of immune (CD45^+^) cells showed that CD45^+^ cells represent a larger population in the LGs of 2M *NOD.H-2b* males compared to age-matched *BALB/c* controls (see material and methods) and *NOD.H-2b* females (**Figures 1A, A’**). The proportion of CD45^+^ cells increased in 6M females compared to 2M females but remained slightly lower than in 6M males (**Figure 1A**). We also evaluated the proportion of B cells within the CD45^+^ cell population using an antibody targeting CD19. It has been reported that CD19 is expressed during all phases of B cell development but not in plasma cells (30). While B cells were barely detected in *BALB/c* males (<1%), the overall proportion of CD45^+^/CD19^+^ cells in 2M *NOD.H-2b* males was significantly higher (21% on average) compared to *NOD.H-2b* females (around 3% on average). At the later stage of the disease, both 6M *NOD.H-2b* males and females had a very high proportion of CD19^+^ cells (around 50% of all CD45^+^ cells). The proportion of T cells (CD45^+^/CD3^+^ cells) was also significantly higher in 2M *NOD.H-2b* males (21%) compared to BALB/c controls (8%) and *NOD.H-2b* females (10%). Males at the early and late stages of the disease (2M and 6M of age) had on average around 20% CD45^+^:CD3^+^ cells, while proportion of the CD45^+^:CD3^+^ cells remained lower in females at both ages (around 10% and 14%, respectively) (**Figure 1A**). The percentage of CD4^+^ and CD8^+^ cells of total cells at 2M was low in BALB/c males (2% and 1% respectively) and *NOD.H-2b* females (3% and <1% respectively) but was significantly higher in *NOD.H-2b* males (14% and 4% respectively) (Figure B,B’). At 6M of age, the percentage of CD4^+^ and CD8^+^ cells in the LGs of *NOD.H-2b* males (14% and 6% respectively) was higher than in 6M females (8% and 2% respectively) (**Figures 1B, B’**). This data suggests that the *NOD.H-2b* females also develop the disease at 6M and the difference in LG inflammation between males and females decreases.

Progressive immunological changes and physical functions decline in healthy mice starting as early as 6M regardless of the mouse strain (31, 32), suggesting that the *NOD.H-2b* females at 6M and older could be also affected by signs of aging.

Comparison of the LG sections of 4M *NOD.H-2b* females (Fid. 1C) and males (**Figure 1D**) (stage not affected by aging) showed that infiltration foci are rare and smaller in female LGs, whereas in the LGs of males, infiltrations are much larger, scattered throughout the whole gland. Most of female LGs had a focus score <1; whereas the focus score for males was above 3 (focus score/4mm^2^, **Figure 1E**). At this stage of the disease the LGs of males showed dilated ducts and numerous vacuoles in the cytoplasm of acinar cells, while the LGs of females were relatively normal (See zoomed image in **Figures 1C, D**). The rounded shape of these vacuoles in the LGs of males suggests an accumulation of lipid droplets in acinar cells (**Figure 1D**), which is consistent with previous reports about diabetic NOD mice (33). This data suggests that the *NOD.H-2b* mouse strain develops LG inflammation in both sexes, however males form the first lymphocytic infiltrations earlier at 2M, while females first form them only around 4M. Therefore, we analyzed the LG transcriptome of the male mice because they develop the disease earlier, which allows us to study LGs unaffected by aging.

### Transcriptomic analysis of the LGs of NOD.H-2b mice reveals inflammatory response and alteration of metabolic pathways

**Figure 2:**
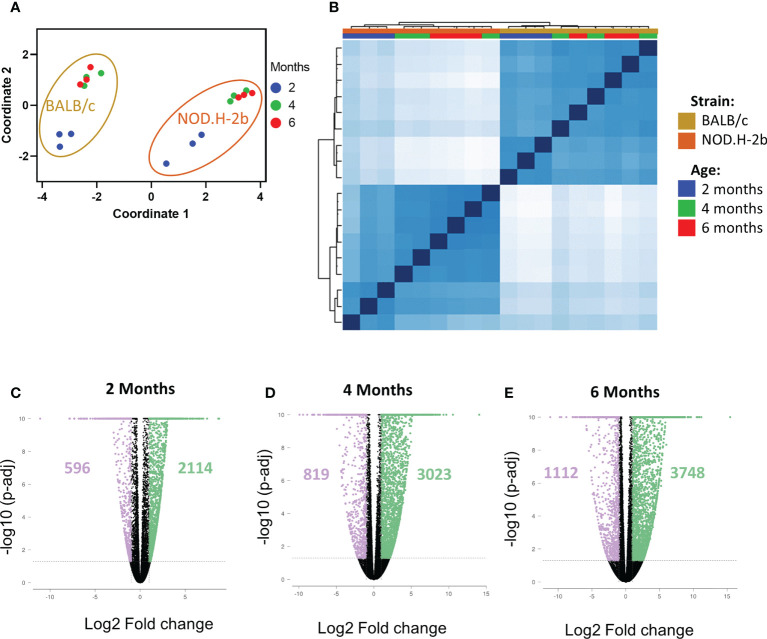
QC of RNAseq samples from NOD.H-2b and BALB/c mice and DEGs. Bulk RNA of LG from NOD.B10 and BALB/c have been sequenced (n=3 per group) at 2, 4 and 6 months. Multidimensional scaling **(A)** and correlation heatmap **(B)** of the different samples sequenced from NOD (in gold) and BALB/c (in orange) at 2 (blue), 4 (green) and 6 (red) months. The DEGs at each stage for NOD.B10 vs BALB/c were defined with an absolute value of FC > 2 with a p-value adjusted < 0.05. Number of DEGs are represented on the volcano plot at 2 months **(C)**, 4 months **(D)** and 6 months **(E)**. The DEGs downregulated are in purple and the DEGs upregulated are in green.

To investigate chronic inflammation development in SS, we performed bulk RNA-sequencing of LGs from *NOD.H-2b* and *BALB/c* (control) males at 2M, 4M and 6M in triplicates. As expected, multidimensional scaling of different samples (**Figure 2A**) and sample correlation heatmaps (**Figure 2B**) showed that *BALB/c* and *NOD.H-2b* data sets form 2 distinct clusters highlighting the inherent differences of their transcriptomics profiles. In each strain, the data from 4M and 6M mice cluster together whereas the 2M groups form a separate cluster, suggesting that there is no major difference between 4M and 6M old LGs whereas there is a major shift in gene expression between 2M and 4M old LGs.

We first identified all differentially expressed genes (DEGs) between *BALB/c* and *NOD.H-2b* mice at 2, 4 and 6 M with a fold change of at least ± 2 and a False Discovery Rate (FDR) inferior to 0.05. Comparison of *NOD.H-2b* with *BALB/c* LGs at 2M (**Figure 2C**) identified 2710 DEGs with 596 genes downregulated and 2114 genes upregulated in *NOD.H-2b* mice. At 4M (**Figure 2D**) this analysis identified 3842 DEGs with 819 genes downregulated and 3023 genes upregulated in *NOD.H-2b* mice. At 6M (**Figure 2E**) this analysis identified 4860 DEGs with 1112 genes downregulated and 3748 genes upregulated in *NOD.H-2b* mice (**Supplemental File 1**).

**Figure 3:**
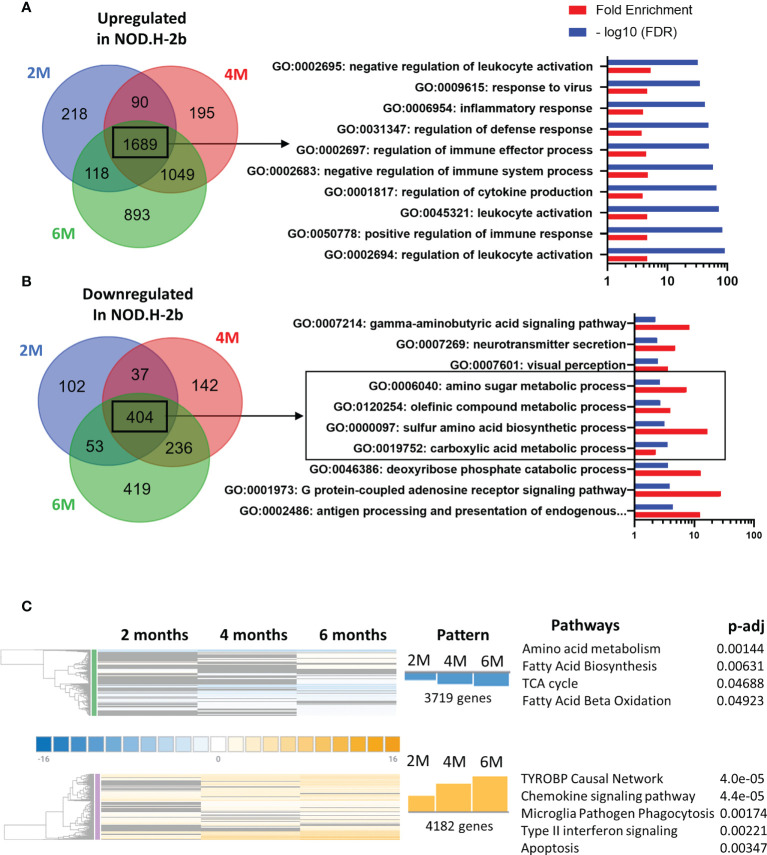
Pathways enriched in DEGs in NOD mice compared to BALB/c at the different ages. Identification of pathways enriched in DEGs common at each stage on the Venn diagram. Common DEGs upregulated **(A)** in NOD.H-B2 mice are exclusively involved in immune system whereas DEGs downregulated **(B)** common at each age are relative to the metabolism. **(C)** Meta-analysis of DEGs revealed two different patterns. In blue, 3719 genes present a pattern with a more important downregulation with age are associated with metabolism. In Orange, 4182 genes present a similar pattern with an increase of their expression from 2 to 6 months. These genes are involved in different type of immune response and apoptosis.

We first analyzed major pathways altered in the pSS mouse model by selecting only genes that have been significantly altered at all ages: thus, 1689 genes were significantly upregulated at 2, 4 and 6 months (**Figure 3A**) whereas 404 gene were significantly downregulated (**Figure 3B**).

Using the Gene Ontology database, we noted that, as expected, the top 10 upregulated pathways were related to inflammatory response and leukocyte activation (**Figure 3A**), which is consistent with appearance of lymphocytic infiltrations in the LG of *NOD.H-2b* males. Downregulated pathways were significantly enriched in DEGs related to “antigen processing and presentation of endogenous peptide antigen *via* MHC class I”, “signal transduction trough G-protein-coupled receptors”, “the metabolism of amino acids”, “neurotransmission”, and several metabolic pathways (**Figure 3B**). The neural dysfunction and impaired neurotransmitter release during chronic inflammation of the LG has been previously reported (34). We also noted the downregulation of several metabolic pathways (See square on **Figure 3B**). These findings correlate with the recently published data on the RNA sequencing of the *NOD.H-2b* mouse submandibular glands (25) and with analysis of diabetic NOD mouse model (33). Taken together our data suggests an altered lipid homeostasis during LG inflammation.

To identify pathways associated with the disease progression, we performed a meta-analysis on gene comparisons between *NOD.H-2b* and *BALB/c* LGs at each age. We identified two major patterns of gene expression correlating with the disease progression (**Figure 3C**). The first pattern of gene expression corresponds to downregulation of genes (**Figure 3C** in blue) which all were involved in metabolic pathways and energy production, such as “amino acid metabolism”, “fatty acid biosynthesis”, “TCA cycle” and “fatty acid beta oxidation” (WikiPathways), suggesting that inflammation of the LG alters mitochondrial function and processing of biomolecules (amino acids and lipids). The second pattern of gene expression (**Figure 3C** in orange) corresponds to pathways significantly upregulated by chronic inflammation. These pathways were mainly involved in inflammatory response and cell death: “TYROBP causal network”, “chemokine signaling pathway”, “microglia pathogen phagocytosis pathway”, “type II interferon signaling”, and “apoptosis” suggesting that inflammation becomes more severe with time. The “TYROBP causal network” pathway has never been previously described in pSS models. *Tyrobp* gene encodes a transmembrane signaling polypeptide which contains an immunoreceptor tyrosine-based activation motif (ITAM) in its cytoplasmic domain. Recently, *Tyrobp* gene was found on a top of the list of diagnostic hub genes in human osteoarthritis (35). The DEGs from the “TYROBP causal network” pathway included macrophage/myeloid markers (*C1qc* and *Itgax* that encodes CD11c) and “microglia pathogen phagocytosis pathway” suggesting increased phagocytosis with disease progression.

### Visium spatial transcriptomics analysis reveals changes in cell subpopulations

Although bulk RNA-seq provides global and comprehensive analysis of the whole LG inflammation, we could not assess the contribution of different cellular compartments in gene expression changes. To combine the benefits of histological analysis with the massive throughput of RNA sequencing, we performed Visum spatial gene expression analysis of 4M *NOD.H-2b* and *BALB/c* LGs sections. Two sections of each strain were sequenced and data from all cryosections were integrated and processed for unsupervised clustering.

To gain more information on transcriptome, one section of each strain was sequenced deeper (BALB/c_2 and NOD.H-2b*_*1). The number of reads and genes detected in *BALB/c* and *NOD.H-2b* cryosections is shown in **Supplemental Figures 1A, B**, and **Table 1**. We noted that areas corresponding to the infiltration foci in the *NOD.H-2b* cryosections were associated with lower number of reads and genes detected (**Supplemental Figures 1A, B**, black arrows). This finding could be explained by the fact that LG epithelium is mainly composed of acinar cells, which have a high RNA content due to their secretory function, while immune cells within foci have lower RNA content. As expected, the number of genes and reads was higher in sections of BALB/c_2 and NOD.H-2b_1 sequenced deeper (**Supplemental Figures 2A, B**; **Table 1**).

**Table 1:** QC metrics of Visium sample.

	BALB/c_1	BALB/c_2	NOD.H-2b_1	NOD.H-2b_2
**Number of spots**	1,135	1,106	1,530	1,505
**Number of reads**	49M	380M	288M	101M
**Mean Reads per Spot**	43,487	343,286	188,827	67,466
**Median Genes per Spot**	874	2,227	1,848	1,051

**Figure 4:**
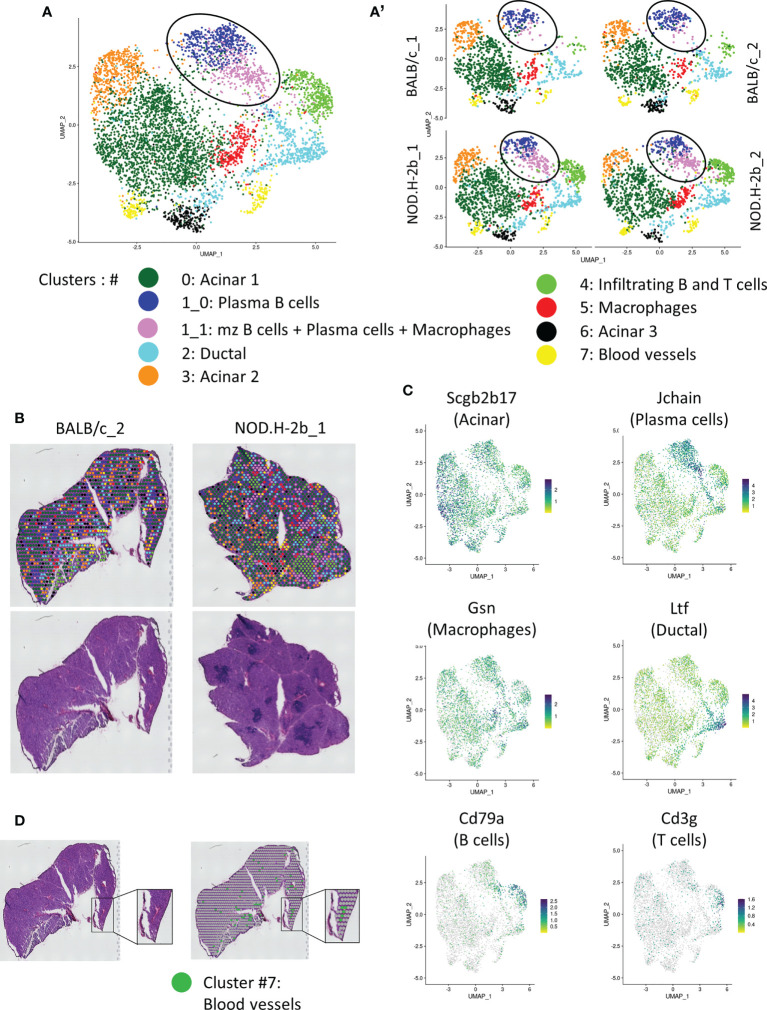
Spatial transcriptomic analysis of LG section from NOD.B10 and BALB/c mice. **(A)** UMAP of integrated samples (2 BALB/c and 2 NOD.H-2B) from 10μm LG cryosections. **(A’)** UMAPs split by samples showing that all clusters are represent in each sample. **(B)** H&E staining of the sections sequenced with spatial localization of the spot colored depending on their clusters. **(C)** Features plot of specific markers defining major clusters: Scgb2b17 (Acinar, Cluster 0, 3 and 6), Jchain (Plasma cells, cluster 1), Cd79a (B cells, cluster 4), Cd3g (T cells, cluster 4), Gsn (Macrophages, cluster 5) and Ltf (Ductal, cluster 2). **(D)** Colocalization of spot from cluster 7 (Erythroid cells) with blood vessels.

Unsupervised clustering using Seurat revealed 8 major clusters (annotated from 0 to 7) (**Figures 4A, B**): clusters enriched with epithelial cells (Clusters #0, 2, 3 and 6) and clusters enriched in stromal cell markers (Clusters #1, 4, 5, and 7) (**Supplemental File 2**, **Supplemental Figures 2**, **3**).

Adult functional LGs are represented by a large epithelial tree, that consists of acini formed by secretory acinar and myoepithelial cells (MECs) (36), connected with ducts and surrounded by stromal cells. MEC signature (*Acta2, Cd109, Epcam, Pax6, Aqp1, Krt5, Krt14, Fgf2*) was found in all clusters (not shown). Indeed, MECs are disseminated throughout the LG tissue since they have very long processes. All acinar clusters (#0, 3, and 6) were enriched in different secretoglobins (*Scgbs*). SCGBs are small, multimeric proteins secreted by epithelial cells of salivary and LG and have been detected in saliva and tears (37, 38). *Scgb2b17* was found in all acinar clusters (**Figure 4C**, **Supplemental Figure 3**). The largest acinar cluster 0 was also enriched in *Scgb2b12, Scgb2b3, Scgb2a2 and Scgb2b18*. Cluster 3 was marked by *Scgb2b3, Scgb2a2, Scgb2b12, Scgb2b18, Scgb2b17* and exocrine gland secreting peptides (*Esp1, Esp15, Esp31*). Cluster 3 was also enriched in the odorant binding proteins (*Obp1a*) and (*Obp1b*) mRNAs. In the LG, the OBP1a protein was found to be a specific target for autoantibodies in SS mouse models (39). Cluster 6 was labeled with expression of carbonic anhydrase VI (*Car6*), several *Scgbs* (*Scgb2a2, Scgb2b12, Scgb2b19, Scgb2b27*), mRNAs of exocrine-gland-secreting peptides (*Esp15 and Esp31*) and mRNA of the mucin like protein-2 (*Mucl2*) (40). Cluster 6 also expressed mRNA of *Mup4* and *Mup5* proteins found only in a subset of LG acinar cells (41).

Ductal cluster 2 was highly enriched in genes known to be expressed in ducts: *Wfdc18, Krt7*, epithelial markers (*Krt8, Krt18*, *Epcam*), and lactotransferrin (*Ltf*) (**Figure 4C**). *Ltf* has been recently found in the LG ducts (42). One of the top markers for cluster 2 was the Deleted in Malignant Brain Tumors 1 (*Dmbt1*) gene encoding a major salivary glycoprotein agglutinin. As expected, ductal cluster was marked with mitochondrial ATPases: *Atp1a1, Atp1b1, Atp1b3, Atp11a, Atp6v1g1, Atp5a1, Atp5e*, and the ATPase Inhibitory Factor 1/IF1 (*Atpif1*), thus supporting the idea that the cells in this cluster are involved in energy metabolism and ATP production for pumps and transporters. We also found some markers of γδT cells, suggesting that ducal cells may be closely associated with this type of T cells. Since current Visium technology does not provide single cell resolution but rather offers resolution of small closely associate groups of cells we also found some acinar markers, such as Aqp5 (43, 44) in this cluster. This may be explained by the close proximity of ductal and acinar cells; since intercalated ducts are directly connected with acini (45).

Four clusters (#1, 4, 5 and 7) were enriched in stromal cells. As the shape and distribution of cluster #1 appeared different between *BALB/c* and *NOD.H-2b* LGs (**Figure 4A**, see circle), we performed sub-clustering that identified cluster (1_0) that was present in both strains, and cluster (1_1) that was more prominent in the *NOD.H2b* LGs. Cluster (1_0) was disseminated throughout LG sections and mainly enriched in plasma B cells, marked by the expression of *Jchain, Igha, Igkc, Iglv1* and *Iglc2.* By contrast, cells from cluster (1_1) surrounded immune foci and were enriched in marginal zone B cells (marked by the *Mzb1*, B and B-1 cell-specific ER-localized protein) (46), some plasma cells (marked by *Jchain*) (47, 48) and macrophages/granulocytes (marked by *Cd74*, *Lyz2* encoding LyzM protein) (**Supplemental File 2**) (49). This finding suggests that these cell types may accumulate at the periphery of the lymphocytic infiltrations. To determine the difference between these two subclusters, we performed gene enrichment analysis compeering cluster (1_1) vs (1_0) of the upregulated (73 genes) and downregulated (71 genes) DEGs (**Supplemental Figure 4**; the list of DEGs is provided in **Supplemental File 3**). Pathways enriched in upregulated DEGs in cluster (1_1) were all related to immunological processes, suggesting that cluster (1_1) contains activated immune cells. While pathways enriched in downregulated DEGs in cluster (1_1) were related to protein export and localization, detection of stimulus and metabolism process. This correlated with the presence of exocrine gland secreting peptides and *Scgbs* in the cluster (1_0). This finding suggests that cluster (1_0) encompassed some epithelial cells that could be associated with plasma B cells which synthesize a polymeric IgA transported into the acinar cells (50, 51).

Cluster 4 was represented by infiltrating B (marked by *Cd79a*) (52, 53) and T cells (marked by Cd3) (54) that colonize the LG during adaptive immune response (**Figures 4A–C**; **Supplemental Figure 3**). Cluster 4 is also marked by expression of inflammation marker *Ctss* which allows MHC II to represent antigen to CD4^+^ T-cells (55) (**Supplemental Figure 3**). Cluster 4 was also marked by macrophage marker *Lyz2* (**Supplemental File 2**). Interestingly, this cluster was almost absent in the LG of BALB/c mice (**Figure 4A’**, **Table 2**) and represented majority of with immune infiltrates within the NOD.H-2b LG section (**Figure 4B**). The infiltrating B cells may differentiate either into memory B cells or plasma cells. Additionally, they behave as antigen-presenting cells and participate in T cell activation during cellular immune responses (56). Cluster 5 was mainly enriched in genes expressed by fibroblasts and/or macrophages (*Apoe, Col3a1, Gsn*, *Ccl8, Ctsb, C1qa*) (57–59). However, this cluster did not have other fibroblasts markers, such as *Col1a1, Pdgfra or Vim*.

**Table 2:** Repartition of spot per cluster and per sample.

	#0	#1_0	#1_1	#2	#3	#4	#5	#6	#7	All
**BALB/c_1**	571 (50.3%)	104 (9.2%)	28 (2.4%)	105 (9.3%)	148 (13.0%)	23 (2.0%)	55 (4.8%)	59 (5.2%)	42 (3.7%)	1135 (100%)
**BALB/c_2**	478 (43.2%)	123 (11.1%)	34 (3.01%)	118 (10.7%)	133 (12%)	37 (3.3%)	43 (3.9%)	89 (8.0%)	51 (4.6%)	1106 (100%)
**All BALB/c**	1049 (46.8%)	227 (10.1%)	62 (2.8%)	223 (10.0%)	281 (12.5%)	60 (2.7%)	98 (4.4%)	148 (6.6%)	93 (4.1%)	2241 (100%)
**NOD.H-2b_1**	613 (40.1%)	112 (7.3%)	149 (9.8%)	164 (10.7%)	139 (9.1%)	169 (11.0%)	78 (5.1%)	52 (3.4%)	54 (3.5%)	1530 (100%)
**NOD.H-2b_2**	588 (39.1%)	119 (7.9%)	172 (11.4%)	151 (10.0%)	100 (6.6%)	171 (11.4%)	94 (6.2%)	34 (2.3%)	76 (5.0%)	1505 (100%)
**All NOD.H-2b**	1201 (39.6%)	231 (7.6%)	321 (10.6%)	315 (10.4%)	239 (7.9%)	340 (11.2%)	172 (5.7%)	86 (2.8%)	130 (4.3%)	3035 (100%)
**All**	2250 (42.6%)	458 (8.7%)	383 (7.2%)	538 (10.2%)	520 (9.9%)	400 (7.6%)	270 (5.1%)	234 (4.4%)	223 (4.2%)	5276 (100%)

Cluster 7 was enriched in markers associated to erythroid cell lineage, such as hemoglobin genes (*Hbb-bt, Hba-a1, Hba-a2, Hbb-bs*). Consistently, cluster 7 spots colocalized with blood vessels (**Figure 4D**).

We also determined the proportion of spots for each cluster between all sections, thus we observed that the proportion of spots in cluster (1_1: mzB cells/plasma B cells/macrophages) and 4 (Infiltrating B and T cells) increased in *NOD.H-2b* mice from 2.8% to 10.6% and from 2.7% to 11.2% respectively (**Table 2**), whereas the proportion of acinar cell clusters 3 and 6 reduced compared to BALB/c from 12.5% to 7.9% and from 6.6% to 2.8% respectively. These findings suggest that in the *NOD.H-2b* LG there is a loss of epithelial cell populations which are replaced by immune cells.

### Validation of cell clusters and inflammatory changes in the *NOD.H-2b* LG

**Figure 5:**
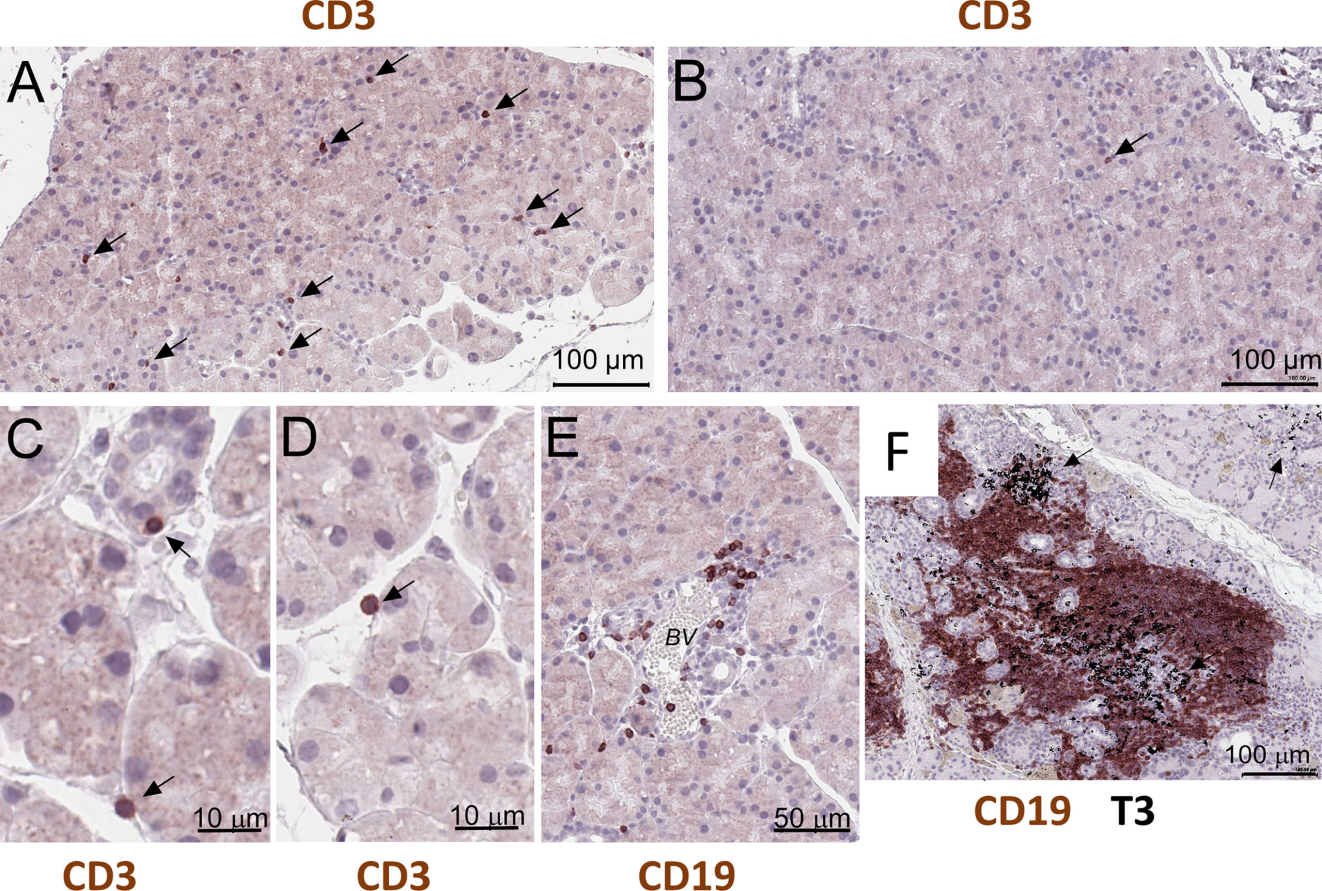
Histological validation of visium alteration. Analysis of T and B cells in the LG of the NOD.H-2b mice. **(A–D)** CD3^+^ cells in the LG of the 2 months old male penetrate throughout tissue and appeared to be attached to duct and acini (black arrows), whereas **(B)** in control BALB/c mice CD3^+^ cells are rare (B, black arrow). **(E)** Later at 3 months of age B cells (stained with the B220 or CD19 antibody) concentrate within an interductular space near the blood vessels (BV). **(F)** Double immunostaining of LG paraffin section with B220 (brown - B cell marker) and T3 (black – T cell marker, black arrows) showing that majority of cells within infiltrate are B cells. B and T cells are organized into an ectopic lymphoid structure.

*NOD.H-2b* mice display reduced lacrimal and salivary gland secretion, formation of mononuclear lymphocytic infiltrations (11, 24), and the presence of SSA and SSB antibodies (21). Immunostaining of paraffin sections of *NOD.H-2b* male LGs with the CD3 (pan T cell marker) antibodies showed that single T cells were scattered throughout whole LG tissue at 6-8 weeks of age in males (**Figure 5A**, black arrows), while in the LGs obtained from BALB/c mice these cells were rare (**Figure 5B**, black arrow). These CD3^+^ cells in the LG of *NOD.H-2b* mice were often found attached to the epithelial ducts and/or acini (**Figures 5C, D**) that partially explains detection of immune cell markers in epithelial clusters. Analysis of B cells immunostained with the CD45R antibody (also known as B220 antibody) showed that initial B cell infiltrates within the LGs appear in the intraductal space near the blood vessels (**Figure 5E**) in the *NOD.H-2b mice* around 2M of age. Double immunostaining of LG paraffin section with B220 (brown - B cell marker) and CD3 (black – T cell marker, black arrows) antibodies shows that the majority of cells within the infiltrates are B cells, although T cells are also present (**Figure 5F**). This supports our Visium finding, that cluster 4 colocalize with immune infiltrates and mainly consists of B cells and the size of this cluster is significantly larger in *NOD.H-2b* compared to *BALB/c* mice (**Figure 4A’**, **Table 2**).

### Spatial transcriptomics identifies pathways altered in all clusters of *NOD.H-2b* LG

**Figure 6:**
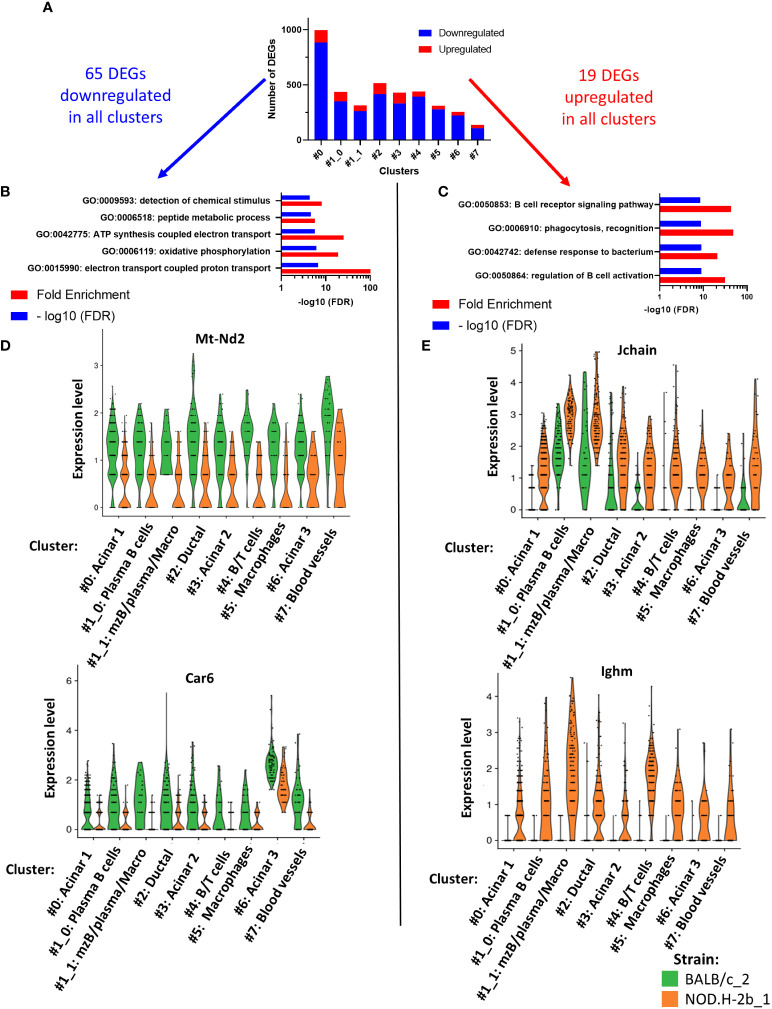
Pathways analysis of DEGs common to all cluster. **(A)** Bar plot showing number of DEGs upregulated (in red) and downregulated (in blue) per cluster. Within all DEGs, 65 were commonly downregulated in every cluster and 19 DEGs commonly upregulated in every cluster. **(B)** Four pathways are significantly enriched in DEGs downregulated and **(C)** four pathways are significantly enriched in DEGs upregulated. **(D, E)** Violin plot of DEGs involved in the pathways previously identified showing their expression in each cluster and depending on their origin: BALB/c (in green) and NOD mice (in orange).

To identify the DEGs between *NOD.H-2b* and *BALB/c*, we used spatial transcriptomic data of only deeply sequenced samples and quantified all DEGs (up and downregulated) in each cluster (**Figure 6A**). Within all DEGs identified (**Supplemental File 3**), there were 65 genes commonly downregulated (in blue) and 19 genes commonly upregulated (in red) in every cluster (**Figure 6A**). Pathway enrichment analysis demonstrated five pathways enriched in downregulated genes (related to “peptide metabolic process”, “detection of chemical stimulus”, “ATP synthesis coupled electron transport”, “oxidative phosphorylation” and “electron transport coupled proton transport”) (**Figure 6B**) and four pathways enriched in upregulated genes that belonged to “regulation of B cell activation”, “phagocytosis, recognition”, “defense response to bacterium” and “B cell receptor signaling pathway” (**Figure 6C**). Examples of genes involved in downregulated (**Figure 6D**) and upregulated (**Figure 6E**) pathways have been presented as violin plots. Thus, all clusters of *NOD.H-2b* LGs showed a decrease in electron transport pathways, illustrated by expression of the *Mt-Nd2* gene, and in the detection of chemical stimuli, illustrated by the *Car6* gene that is known to regulate pH levels of secreted fluid in the salivary gland (60). The top pathway enriched in upregulated genes, was the “B cell receptor signaling” pathway (**Figure 6C**). This pathway is represented by the expression of *Jchain* (encoding joining chain of multimeric IgA and IgM) and *Ighm* gene (expressed in plasma B cells) (**Figure 6E**). The increase *Jchain* and *Ighm* expression in all clusters suggests that infiltrating cells may be present in the different parts of the LG. The highest increase in *Jchain* and *Ighm* was found in cluster 1 (**Figure 6E**).

Altogether Visium confirms our bulk RNAseq data showing increase in inflammatory response and downregulation of lipid metabolism in the *NOD.H-2b* LG and indicates that these changes affect the entire LG.

### Spatial transcriptomics identifies pathways specifically altered in each cluster of *NOD.H-2b* LGs

**Figure 7:**
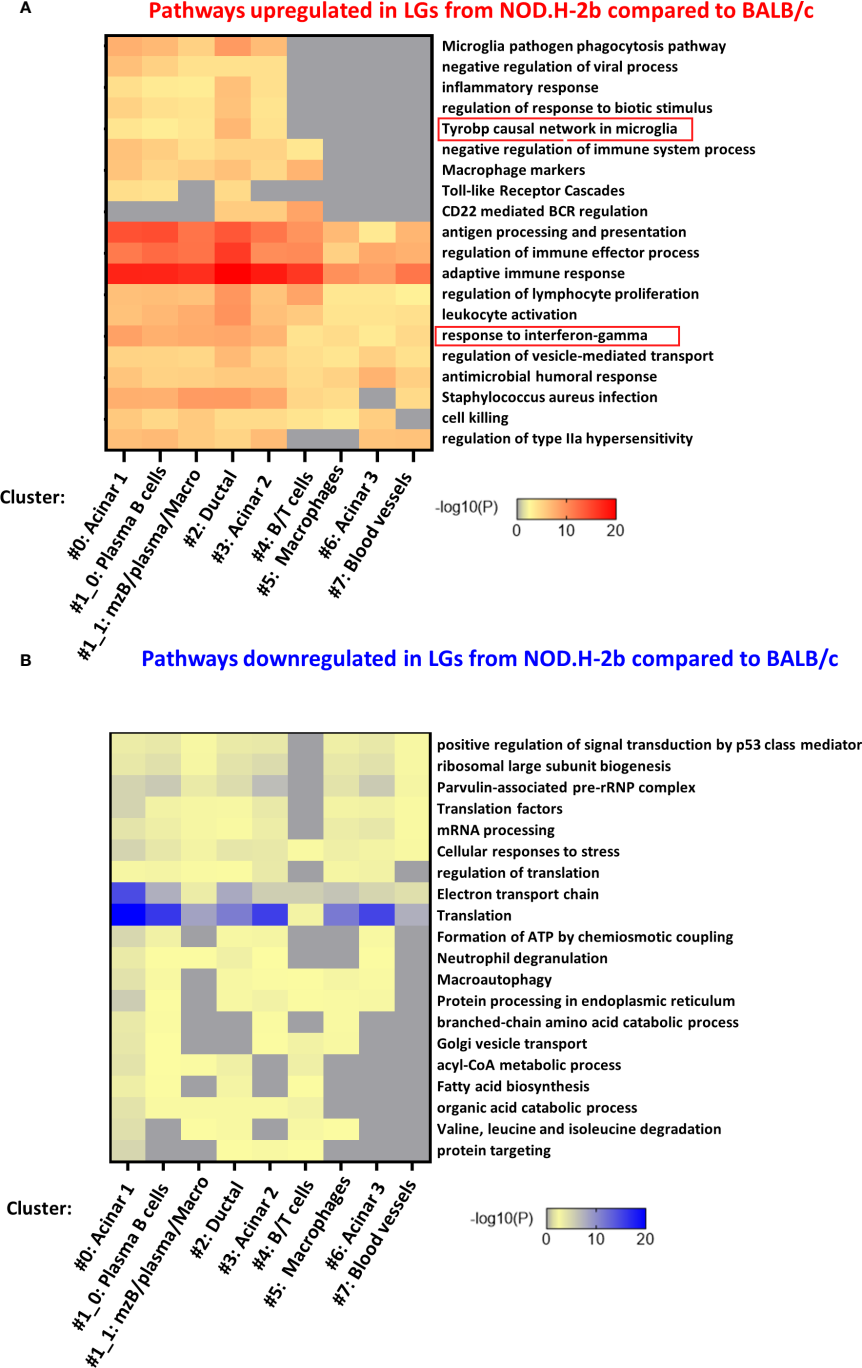
Pathway enrichment in DEGs for each cluster. The 7 lists (one for each cluster) of all **(A)** upregulated and **(B)** downregulated DEGs between NOD.H-2b_1 vs BALB/c_2 samples were submitted on www.metascape.org with default parameter (61) to identify altered pathways the most significantly enriched per cluster. The top 20 of enriched terms are presented on a heatmap based on their adjusted p-value.

To identify specific pathways altered in each cluster, we analyzed the list of DEGs identified in each cluster separately. **Figures 7A, B** shows the top-20 upregulated and downregulated pathways. Almost all of them were found in four clusters 0, 1, 2 and 3 (**Figure 7A**) corresponding to the acinar cell clusters (#0 and #3), ductal cluster (#2), and immune cell cluster (#1, enriched in MZB, plasma cells and macrophages). These pathways were related to the inflammatory processes or activation of immune cells. Eight pathways upregulated in *NOD.H-2b LG* were altered in all clusters while five pathways were altered only in cluster 0, 1, 2 and 3. These four clusters had the highest number of spots within the LG sections. The pathway “Microglia pathogen phagocytosis” also identified by the meta-analysis of bulk RNA-seq data was highly enriched in the ductal cluster 2 (**Figure 7A**). Interestingly, the list of the top 100 pathways showed that the majority of significantly altered biological processes including “response to virus” and TLR signaling were found in clusters #0 (acinar) and #1_0 (plasma and acinar cells) (**Supplemental Figure 5**). Thus, activation of inflammatory response in the LG with disease progression is not specific to immune cells but also present in the LG epithelial clusters (acinar and ductal), suggesting that epithelial cells may participate in primary inflammatory response. It is also possible that activated immune cells could be closely associated with epithelial cells in *NOD.H-2b* LGs (**Figure 5**), since each 55 μm-spot encompasses several cells (**Supplemental Figure 6**). Consistent with this, the “macrophage marker” pathway was upregulated in several epithelial cluster (#0, 2 and 3, **Figure 7A**) and we indeed observed an increased number of spots positive for macrophage marker genes in epithelial clusters of the *NOD.H-2b* LG section (**Supplemental Figure 7**).

Analysis of pathways enriched in downregulated DEGs shows that most of these pathways were also altered in the acinar cluster #0 (**Figure 7B**; **Supplemental Figure 8**). These pathways were “translation”, “protein processing in endoplasmic reticulum”, “electron transport chain”, and related to lipid metabolism (“acyl-CoA metabolic process”, “fatty acid biosynthesis”, “organic acid catabolic process”). This is consistent with the loss of secretory functions and decrease of metabolism in the acinar cells of the *NOD.H-2b* LGs due to cell damage (**Figure 1D**; see vacuolization of acinar cells). “Electron transport chain” and “Translation” pathways were altered in several other clusters (clusters # 1, 2, 3, and 6) (**Supplemental Figure 8**). Mitochondrial dysfunction is widely implicated in aging and chronic inflammation of many tissues (62). Thus, several studies report an important role for mitochondria in cellular homeostasis and show that dysfunctional mitochondria can release components (such as reactive oxygen species) that can act as DAMPs and induce an inflammatory response through activation of the pattern recognition receptors (PRRs) (63, 64). In addition, the clusters with the highest numbers of DEGs associated with “response to interferon-gamma” were epithelial (#0: acinar, #2: ductal, and #3: acinar) (**Figure 7A**). Altogether, these results suggest that the LG epithelium undergoes profound internal metabolic changes, while being challenged by the numerous pro-inflammatory stimuli produced by surrounding activated immune cells.

### Pathways identified by bulk RNA-seq show specific distribution within the LG of *NOD.H-2b* mice

**Figure 8:**
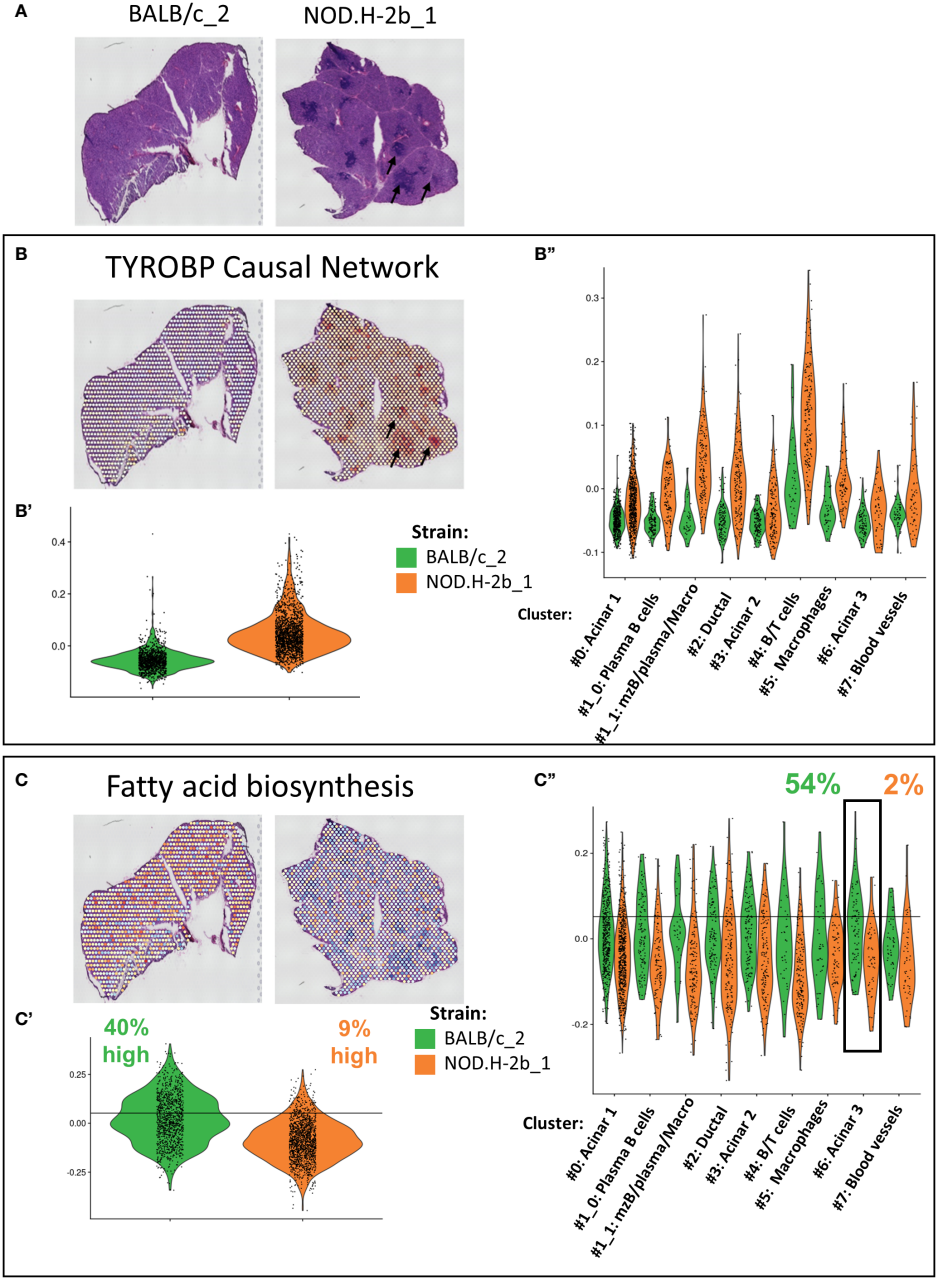
Validation and localization of altered pathways identified by meta-analysis of bulk RNAseq in Visium. **(A)** Scan of cryosections samples sequenced with visium are presented for reference. Genes involved in altered pathways identified by the meta-analysis in **Figure 3C** have been used to established a signature of genes. Score of **(B)** TYROBP Causal Network (upregulated in NOD bulk RNAseq) with Violin plot comparing score between BALB/c and NOD of the whole section **(B’)** and per cluster **(B”)**. **(C)** Fatty acid biosynthesis (downregulated) in NOD bulk RNAseq. signature was calculated for each spot and plot on visium samples with Violin plot comparing score between BALB/c and NOD of the whole section **(C’)** and per cluster **(C’’)**.

Our bulk RNAseq analysis identified several pathways altered over disease progression in *NOD.H-2b* LGs. To localize and eventually identify the cellular compartments contributing to these changes, we calculated the score corresponding to the expression of genes involved in these pathways for each spot (**Figure 8**, **Supplemental Figure 9**). The most significant pathway enriched in upregulated DEGs was the “TYROBP Causal Network”, for which we observed a higher score in the *NOD.H-2b* LG samples (**Figures 8A, B**), particularly around and within infiltration foci (**Figure 8B**, black arrows) where the majority of activated macrophages are present (65, 66). The Violin plots (**Figures 8B’, B’’**) confirms the results of bulk RNAseq and shows that the “TYROBP Causal Network” pathway is highly enriched in the cluster 4 (B and T cells). We also found a higher score for TYROBP signaling in clusters #1_1 (mzB cells/plasma B cells/macrophages). The increase of this score in all clusters of *NOD.H-2b* LGs is consistent with our previous observation showing an increase of macrophage marker in epithelial cell clusters. (**Figure 7A**, **Supplemental Figure 7**).

One of the top downregulated pathways was the “fatty acid biosynthesis pathway”, which was downregulated in *NOD.H-2b* LG (**Figure 3C**). Spots with the highest score for this pathway were evenly distributed throughout the *BALB/c* section, whereas the average score for the *NOD.H-2b* LG was significantly decreased, and was particularly low in spots colocalized with immune foci (**Figures 8C, C’**). To define spots with a high score, we set a threshold corresponding to the value exceeded by 40% of total BALB/c spots. Only 9% of spots in the *NOD.H-2b* LGs were above this threshold (**Figure 8C’**). We also noted that acinar cluster #6 in the *BALB/c* LGs had the highest percentage of spots (54%) with a high score for the “fatty acid biosynthesis pathway, while in the *NOD.H-2b* LGs this cluster had the lowest percentage (2%). This suggests that the acinar cluster #6 has active lipid metabolism in *BALB/c* LGs, while it is decreased in the diseased glands of the *NOD.H-2b*. As we showed above, this acinar cluster-6 expressed *Car6* (**Figure 5B**) that plays an important role in the detection of chemical stimulus and is presumed to have a role in the maintenance of acid/base balance on the surface of the eye, skin, and oral cavity (41).

## Discussion

In this study using bulk RNAseq and Visium spatial transcriptomics approaches, we investigated the molecular mechanisms promoting the development of chronic inflammation in the LGs of *NOD.H-2b* mice. We analyzed the LG transcriptome of 2, 4 and 6M *NOD.H-2b* and *BALB/c* mice LGs by bulk RNA-seq to identify the driver mechanisms/pathways for disease progression and used Visium to evaluate special distribution of cell groups and visualize activation or downregulation of specific pathways found by bulk RNAseq within the LG section. Our bulk RNA seq data of the LG of 2, 4 and 6M old *NOD.H-2b* mice suggests that LG inflammation becomes progressively more severe with the time, while mitochondrial function, along with amino acid and lipid metabolism in epithelial cells decreases. Thus, we found that upregulated genes in the LGs NOD.H-2b mice were related to inflammation and leukocyte activation, which is consistent with activation of the adaptive immune response associated with infiltration of lymphocytes.

The major limitation of Visium is the lack of single cell resolution: each 55 µm spot encompassed several cell types (**Supplemental Figure 6**) and failed to clearly identify small and disseminated cells like MECs or fibroblasts that were mixed with other cell types within one spot. However, the possibility to analyze the whole transcriptome throughout the LG section provided an excellent opportunity to determine which cells are closely associated with each other and enables a high-resolution view of cellular dynamics and interactions. Our study using Visium spatial transcriptomics successfully identified the major cellular components of healthy and chronically inflamed LGs and determined specific clusters/cell types within the LG sections. As expected, we observed an increased proportion of the immune cluster #4 containing infiltrating immune cells and activation of immune response pathways within this cluster. Increased proportion of cluster #4 (composed of infiltrating B/T cells, and macrophages) and the presence of cluster 1_1 (mz B cells/plasma cells/macrophages) specifically in the *NOD.H-2b* LG shows the induction of the adaptive immune response in the *NOD.H-2b* LGs. Moreover, the majority of spots in cluster #4 were positive for B cell markers (*CD79a*) while T cell markers were less frequent. This is consistent with our flow cytometry data and the human data showing that in the LGs of SS patients B cells are present in higher proportion than T cells (67).

Using the bulk RNA-seq data meta-analysis we found that an increasing number of upregulated genes over the course of disease progression were related to macrophage activity and the innate immune response. Indeed, the highest pathways containing upregulated DEGs belonged to the “TYROBP causal network”, “chemokine, type II interferon (IFN II) signaling”, “microglia phagocytosis” and” apoptosis.” Visium data analysis also showed that the “TYROBP casual network” was highly enriched within and around the lymphocytic infiltrations, suggesting that this pathway is activated in immune cells. Since TYROBP protein was specifically identified as a universal biomarker for macrophages (68), our data suggests that activation of the TYROBP casual network” pathway in the macrophages drives the progression of inflammation. Although the “TYROBP casual network” was not reported previously for SS, recent publications reveal that TYROBP is overactivated during other autoimmune diseases, such as Huntington’s, Alzheimer’s disease and osteoarthritis (35, 69–72). Moreover, it was reported that deletion of *Tyrobp* ameliorates neuronal dysfunction/atrophy and reduces pro-inflammatory pathways that are specifically active in human brain during these diseases (69). TYROBP signaling also leads to the production of the IL-18, that facilitates IFN-γ production by NK cells. IFNγ activates IFN II signaling that links the innate and adaptive immune responses (73). In macrophages, IFN-γ promotes phagocytosis and release of reactive oxygen species and nitric oxide (NO) (74). NO reduces blood flow by inducing vasodilatation of blood vessels promoting the extravasation of the recruited immune cells (75). IFN-γ also induces M1 macrophage polarization and priming to secrete pro-inflammatory cytokines including IL-1β, TNF-α and IL-12 (76–78). Thus, during central nervous system autoimmune disease, the IFN-γ and IL-12 promote the differentiation of naïve T cells into the Th1 cells, which are major producers of pro-inflammatory cytokines (79, 80). In addition, in human SS patients the CD4^+^ T cells are mainly the Th1 cells producing IFNγ (81). Interestingly, according to our Visium data, the “response to interferon-gamma” pathway is enriched in the acinar and ductal (#0, 2, 3) clusters (also see **Figure 7A**). Altogether our findings and those of other publications suggest that the IFN-γ-activated macrophages are responsible in the exacerbation of inflammation and epithelial dysfunction over the time of disease progression. Our findings also suggest that decrease of TYROBP expression/activation and/or IFN-II signaling could be beneficial as a treatment of the SS.

One of the most unexpected findings was that activation of the inflammatory pathways was found in all epithelial cell clusters, suggesting that the epithelial compartments participate in the inflammatory process. The contribution of the epithelial cells to disease progression was reported for the SMG of the pSS mouse model and in patients with SS (25, 82). Furthermore, corneal epithelial cells act as an initial physical and immunological barrier to injury/infection playing an important role in the immune response (83). Moreover, a recent study reports a significant activation of the absent in melanoma-2 (AIM2) inflammasome in the ductal LG epithelial cells of SS patients (84). Thus, epithelial Aim2 inflammasome can sense viruses, bacteria, and cell damage and initiate primary immune response in the LG epithelial cells (84).

Another mechanism driving disease progression highlighted by meta-analysis was downregulation of genes involved in amino acid and lipid metabolism and the ATP production by the tricarboxylic acid (TCA) cycle. Our Visium data show that, while the electron transport chain enabling ATP synthase activity is altered in the whole LG, the metabolism of carboxylic acids (including amino acid and lipids) and the whole process of protein synthesis are mostly altered in acinar clusters. Although “fatty acid biosynthesis” pathway and other metabolic pathways were downregulated in all clusters of the *NOD.H-2b* LGs compared to BALB/c LGs, the highest alteration of these pathways was observed in the acinar 3 cluster #6 marked by *Car6* gene expression. Car6 encoded protein carbonic anhydrase 6 (CA-VI) was previously found in acinar cells of the rat, sheep, and human LGs and SMG (41, 85). We found a significant decrease in *Car6* expression associated with fewer *Car6*-positive spots within the *NOD.H-2b* LG sections (also see **Figure 6D**) suggesting the loss and/or dysfunction of the *Car6*-expressing acinar cells. A recent study also reported detection of autoantibodies against CA VI protein in the SS patients (86) which may explain the decrease in *Car6* expression due to damage of *Car6*-expressing cells. Further studies are needed to understand the role of the *Car6* marked subpopulation of LG acinar cells in the SS.

In conclusion, our data show that in the LG of the pSS mouse model activation of the macrophage related processes (TYROBP pathway) and downregulation of the lipid and mitochondrial metabolism in the acinar cells of the LG are the potential driver mechanisms for progression of chronic inflammation. Modulation of these pathways may potentially reduce the inflammatory stimuli in the LG and could be applicable to treat the SS in the future.

### Data and code availability

Raw data from RNA-sequencing of LGs from *BALB/c* and *NOD.H-2b* at 2, 4 and 6M generated for this study have been deposited at the Gene Expression Omnibus GSE210332

Visium spatial gene expression dataset has been deposited at the Gene Expression Omnibus GSE210380. Ready-to-use SEURAT object is also provided as **Supplemental File 4**. Code used to analyze Visium data and generate figures in this article is provided in **Supplemental File 5**.

## Materials and methods

### Experimental model

The pSS mouse model *NOD.H-2b:* Strain #:002591 (RRID : IMSR_JAX:002591) and healthy controls *BALB/c; s*train #:000651 (RRID : IMSR_JAX:000651) mice were purchased from the Jackson Laboratory (Sacramento, CA, USA) and were fed ad libitum and kept under a 12-hour light/12-hour dark cycle. We have chosen *BALB/c* mice as a control mouse strain since they were more comparable to NOD.H*-2b* mice regarding their body weight and LG size (which is important for Visium experiments) than the C57BL10 mice. BALB/c mice are commonly used as healthy controls for similar mouse strain NOD/ShiLtJ and other SS mouse models for lacrimal gland studies (87–89). All experiments were performed in compliance with the ARVO Statement for the Use of Animals in Ophthalmic and Vision Research and the Guidelines for the Care and Use of Laboratory Animals published by the US and National Institutes of Health (NIH Publication No. 85-23, revised 1996) and were preapproved by TSRI Animal Care and Use Committee.

### Lacrimal gland histology and immunostaining

Extraorbital LGs were dissected and fixed in 10% formalin and processed for paraffin embedding in the Scripps histology core facility or by Allele Biotech at San Diego. Five μm sequential paraffin sections were deparaffinized and stained with the CD45R antibody (clone RA3-6B2, #sc-19597, Santa Cruz Biotechnologies; RRID: AB627078), also known as B220 antibody targeting B cells, or rabbit monoclonal CD3 antibody (clone SP7, #NB600-1441, Novus Biologicals; RRID: AB_789102) to visualize T cells as was described previously (90).

Briefly, endogenous peroxidase activity on rehydrated sections was blocked by treating slides with 3% hydrogen peroxide in absolute methanol for 30 mins. Antigen retrieval was performed for 40 mins using 0.01 M citrate (pH 6.0) in a humidified heated chamber. Sections were blocked with 5 g/L casein in PBS containing 0.5 g/L thimerosal (Sigma-Aldrich; cat# T5125-25G) for 30 minutes, incubated with primary antibodies, and diluted in casein buffer overnight at 4°C. CD3 and CD45R antibodies. Appropriate biotinylated secondary antibodies (Vector Labs, Burlingame, CA) were used at a 1:300 dilution. Visualization was achieved using biotin/avidin-peroxidase (Vector Labs) and Nova Red (Vector Labs). Tissue was counterstained with Gill’s hematoxylin (Fisher Scientific, San Diego, CA; CS400). To study distribution of B and T cells in the same infiltration foci two sequential 5 μm sections were stained with CD45R or CD3 and layer of CD3 labeling was overlapped with section stained with CD45R antibody using CANVAS X Draw software (Canvas).

### Focus score

Paraffin sections of LGs stained with hematoxylin and eosin were used to calculate the focus score. The immune foci were defined as mononuclear cell infiltrates containing at least 50 inflammatory cells. The number of foci was divided by the surface area (in mm^2^) of the section and multiplied by 4 to obtain the focus score/4 mm^2^. The area measurements were done with QuPath (91).

### Flow cytometry

For flow cytometry, LGs from young (7-10 week-old) males and from mature adult (28-32 week-old) *NOD.B10-H-2b* males/females were processed individually. While due to small sizes LGs of three young *BALB/c* (7-10 weeks old) males were combined to obtain enough material for flow cytometry. Similarly, LGs of young *NOD.B10-H-2b* (8-13 weeks old) females were combined (each sample contained LGs from 3 mice) to obtain sufficient number of cells for analysis. LGs were dissected, rinsed with ice-cold DPBS (# 14190-144, Gibco) and quickly minced in a dish containing 500 µL of digestion medium [DMEM/F12 (#11-330-032, Fisher Scientific), 0.5% BSA (#SH3057401, Fisher Scientific), 15 mM HEPES (#MT25060CI, Fisher Scientific), 3 mM CaCl_2_ (#501035464, Fisher Scientific), 125 U/mL collagenase IV (#C5138, Sigma), 20 U/mL DNase-I (#DN25, Sigma)]. Cell suspension was transferred into a gentleMACS C tube (#130-093-237, MACS Miltenyi) and fresh digestion medium was added up to 2 mL total. The following programs were successively used with the gentleMACS Octo Dissociator (#130-096-427, MACS Miltenyi): “37C_m_TDK_1”, “m_impTumor_02”, “15 min incubation (37° spin ±20 rpm every 10 min)”, “m_impTumor_02 for a total digestion time of 1 h”. At the end of the digestion, samples were gently triturated by pipetting up and down 15 times with a 1 mL pipet tip, cells were pelleted (300 x g, 5 min, 4°C), washed with ice-cold blocking medium [HBSS 1X, 0.5% BSA, 10 mM HEPES, 1 mM EDTA (#AM9260G, Fisher Scientific)], pelleted again (300 x g, 5 min, 4°C), and treated with the DNAse (HBSS 1X, 0.5% BSA, 10 mM HEPES, 2 mM CaCl_2_, 2 mM MgCl_2_ (#AM9530G Fisher Scientific), 200 U/mL DNase-I) for 10 min at RT. The cell suspension was then sequentially passed two times through a 3 mL syringe with needles: 18G, 22G and 25G and LG cells were filtered through the 70-µm mesh cell strainer and pelleted by centrifugation (400 x g, 5 min, 4°C). As an additional control we used spleen obtained from the same mice. Spleens were dissociated with the spleen program: “m_spleen_01” in C-tubes filled with blocking medium spleen cells were sequentially filtered through the 100-µm and 70-µm cell strainer (15-1070; 15-100; Biologix USA) and pelleted by centrifugation (400 x g, 5 min, 4°C).

Erythrocytes were removed by incubation in the RBC lysis buffer 1X (#5831-100, Biovision); 5 min incubation at RT and cells were pelleted by centrifugation (400 x g, 5 min, 4°C). Pellets were resuspended in 2 mL of FACS buffer (DPBS, 0.5% BSA, 25 mM HEPES, 1 mM EDTA) and the number of viable cells was estimated trypan blue (#1691049, Fisher Scientific, San Diego, CA) staining analyzed by the Countess 3 FL (ThermoFisher Scientific). Cells were pelleted (400 x g, 5 min, 4°C) and incubated with the anti-mouse CD16/32 (#101320, Biolegend) on ice for 5 min to block non-specific binding of immunoglobulins to the Fc receptors. Then, cells were incubated on ice for 45 min with the following antibodies: (1) CD4-FITC (#100406, Biolegend) and CD8-PE (#100707, Biolegend) or (2) CD45-FITC (#103112, Biolegend), CD3ϵ-BV605 (#100351, Biolegend) and CD19-APC (#115512, Biolegend). After antibodie(s) treatment cells were washed and resuspended with FACS buffer. A viability dye (FxCycle Violet stain, #F10347, Molecular probes by Life Technologies) and nuclear specific DRAQ5™ (#4084S, Cell Signaling Technology) were added to discriminate dead/live intact cells. Samples were analyzed at the TSRI Flow Cytometry Core Facility. The main population of live lymphocytes was determined by forward scatter (FSC) and side scatter (SSC) area gating and by dead cell exclusion. Doublets were excluded *via* FSC-Area vs Width and SSC-Area vs SSC-Width gating. Unstained and single-color controls were prepared for each experiment to determine the background noise and spleen samples were used as positive controls to gate cells of interest. Data analysis was performed using FlowJo v10 software.

### Bulk RNA sequencing and data analysis

LGs of *NOD.H-2b* and BALB/c males at 2, 4 and 6 months of age were dissected and processed for RNA isolation and RNA sequencing. Each sample corresponded to one LG obtained from one mouse and each group consisted of 3 animals. After the LG was placed in cold PBS, the capsule and surrounding tissues were removed under the microscope. The LG was washed two times in cold PBS and placed into 150 µL tissue RNA later solution (AM7020 Ambion by Invitrogen). When all LGs were collected, RNA later was removed, and each LG was transferred into 2mL bead beating tubes (cat# 19-628, OMNI, Kennesaw GA) containing 700 µL of Qiazol lysis reagent (#79306, Qiagen). The LG was disrupted using Bead Ruptor 4 (cat# 25-010, Omni) in two cycles: first at speed 5, for 40 sec and then at speed 5, for 30 sec. Between these two cycles, the tubes were placed on ice for 2 mins. RNA was isolated with the miRNeasy Mini kit (#217084, Qiagen), according to the manufacturer’s instructions.

The amount of total RNA was estimated by a Nanodrop ND-1000 spectrophotometer and checked for purity and integrity (RIN) in a Bioanalyzer-2100 device (Agilent Technologies, Inc., Santa Clara, CA). 800 ng of total RNA (RIN>8) from each sample was used to prepare RNAseq libraries using the NEBNext rRNA Depletion Kit (Human/Mouse/Rat) followed by the NEBNext Ultra II RNA Library Prep Kit for Illumina (9 cycles of PCR). Completed libraries had been sequenced (2 x 75 paired ends) using Illumina NextSeq500 to generate 50M reads for each sample.

Data was analyzed using ROSALIND^®^ software (https://rosalind.onramp.bio/), with the HyperScale architecture package developed by ROSALIND, Inc. (San Diego, CA). Reads were trimmed using cutadapt (92). Quality scores were assessed using FastQC2. Reads were mapped against the mouse Mus musculus genome reference (GRCm38 genome index for analysis was built using STAR (93). With at least 49 million reads per sample, the percentage of post-trimming reads that aligned to the reference genome for each sample was > 97%. Individual sample reads were quantified using HTseq (94) and normalized *via* Relative Log Expression (RLE) using DESeq2 R library (95). Read distribution percentages, violin plots, identity heatmaps, and sample MDS plots were generated as part of the QC step using RSeQC (95). DEseq2 was also used to calculate fold changes and p-values and perform optional covariate correction. Clustering of genes for the final heatmap of differentially expressed genes was done using the PAM (Partitioning Around Medoids) method using the fpc R library (https://cran.r-project.org/web/packages/fpc/index.html). Hypergeometric distribution was used to analyze the enrichment of pathways, gene ontology, domain structure. The topGO R library, was used to determine local similarities and dependencies between GO terms to perform Elim pruning correction. Several database sources were referenced for enrichment analysis, including Interpro (96), NCBI (97), MSigDB (98), REACTOME (99), and WikiPathways (100). Enrichment was calculated relative to a set of background genes relevant for the experiment.

### Visium spatial gene expression experiments

Optimal parameters for tissue permeabilization for the LG were determined using Visium Spatial Tissue Optimization reagents according to the manufacturer’s instructions (10x Genomics, Pleasanton, CA, USA). Visium Spatial Gene Expression was then carried out according to the manufacturer’s instructions (10x Genomics, Pleasanton, CA, USA) with the following parameters: 10μm thick sections of LG were used, hematoxylin and eosin (H&E) staining was carried out by incubating slides for one minute in isopropanol, 7 min in hematoxylin and 1min in eosin. Permeabilization was carried out for 18 min.

All Visium libraries were sequenced simultaneously on the Illumina NextSeq2000 platform, at a sequencing depth of approximately 49M read-pairs for BALB/c_1, 52M for BALB/c_2, 85M for NOD.H-2b_1 and 100M for NOD.H-2b_2. To gain more insight information, a second round of sequencing was performed for BALB/c_2 and NOD.H-2b_1, which finally reached 380M and 288M read-pairs respectively. Fastq files were processed with spaceranger-1.3.1 pipeline. Then processed data was analyzed on R studio (2021.09.1, Build 372) with Seurat (v4.0.4)

### Visium spatial gene expression analysis

Fastq files were processed with spaceranger-1.3.1 pipeline. Then, processed data were analyzed on R studio (2021.09.1, Build 372) with Seurat (v4.0). Quickly, data from the four samples were merged and normalize with SCTransform function. Then data were integrated, and Uniform Manifold Approximation and Projection (UMAP) was generated with a resolution of 0.42. Cell type marker lists were generated to identify the different clusters. For DEGs identification we used the “FindMarker” function only on deeply sequenced samples (BALB/c_2 and NOD.H-2b_1). Finally, to confirmed alteration observed with meta-analyses on bulk RNAseq, we established gene lists from ROSALIND^®^: we selected all altered genes from pathways identify by the meta-analyses. Then, expression of these pathways was assessed on Visium with the function “AddModuleScore” and values for each spot were plot on Visium section.

### Biological pathway analysis

From the list of differentially expressed genes DEGs identified by RNAseq, we used Gene Ontology database (http://geneontology.org/) to identify the altered biological pathways. To achieve this, we submitted the lists of DEGs and performed a GO enrichment analysis. Then, we selected only the top-10 of biological pathways in which we had at least 10 genes altered and the FDR inferior to 0.05. Heatmaps of significantly altered pathways were generated using www.metascape.org with a default parameter (61).

### Statistical analysis

Flow cytometry: Average values are shown as mean ± SD. To compare multiple groups, a one-way ANOVA was performed when data follow a normal distribution (Shapiro-Wilk test; p-value > 0.05). For CD4^+^ comparison, data does not follow normal distribution (Shapiro-Wilk test; p-value = 0.0363 for Young Male BALB/c), a Kruskal-Wallis test has been performed A p-value <0.05 was considered statistically significant. P-value significance: *: p <0.05; **: p <0.01; ***: p <0.001; ****: p <0.0001. All statistical analyses were performed in GraphPad Prism (v9.3.0).

Focus score: Values are shown as mean ± SD. Shapiro-Wilk test has been performed to determine that data follow normal distribution (p-value >0.05). Statistical significance between male and female has been assessed with an unpaired t-test (**: p=0.0021).

## Data availability statement

The datasets presented in this study can be found in online repositories. The names of the repository/repositories and accession number(s) can be found below: https://www.ncbi.nlm.nih.gov/, GSE210332; https://www.ncbi.nlm.nih.gov/, GSE210380.

## Ethics statement

All experiments were performed in compliance with the ARVO Statement for the Use of Animals in Ophthalmic and Vision Research and the Guidelines for the Care and Use of Laboratory Animals published by the US and National Institutes of Health (NIH Publication No. 85-23, revised 1996) and were preapproved by TSRI Animal Care and Use Committee.

## Author contributions

OM: Conceptualization, Data curation, Formal analysis, Investigation, Methodology, Software, Validation, Writing – original draft, Writing – review and editing. VD: Conceptualization, Data curation, Formal analysis, Investigation, Methodology, Validation, Writing – original draft, Writing – review and editing. TU and CP: Methodology, Validation, Writing – review and editing. DD: Conceptualization, Funding acquisition, Methodology, Validation, Visualization, Writing – review and editing. HM: Conceptualization, Data curation, Formal analysis, Funding acquisition, Investigation, Methodology, Project administration, Resources, Supervision, Validation, Visualization, Writing –original draft, Writing – review and editing. All authors contributed to the article and approved the submitted version.

## Funding

This work was supported by the research grants from the National Institute of Health, (NIH), National Eye Institute (NEI), United States, Grant 5R01EY026202 (HPM and DAD), EY030447 (CSdeP) and National Institute of Dental and Craniofacial Research (NIDCR) grant R01DE031044 (HPM), unrestricted grant from Research to Prevent Blindness.

## Acknowledgments

We are grateful to Dr. Steven Head and Dr. Tony Mondala and the Scripps Research Institute Genomics Core Facility for performing sequencing and to Dr. Scott Henderson for scanning Visium sections.

## Conflict of interest

The authors declare that the research was conducted in the absence of any commercial or financial relationships that could be construed as a potential conflict of interest.

## Publisher’s note

All claims expressed in this article are solely those of the authors and do not necessarily represent those of their affiliated organizations, or those of the publisher, the editors and the reviewers. Any product that may be evaluated in this article, or claim that may be made by its manufacturer, is not guaranteed or endorsed by the publisher.
