# Supplementary figures and images for: Spatial transcriptomics of the lacrimal gland features macrophage activity and epithelium metabolism as key alterations during chronic inflammation
Source: Front Immunol. 2022 Oct 17;13:1011125. doi: 10.3389/fimmu.2022.1011125 (PMC9628215; doi:10.3389/fimmu.2022.1011125)

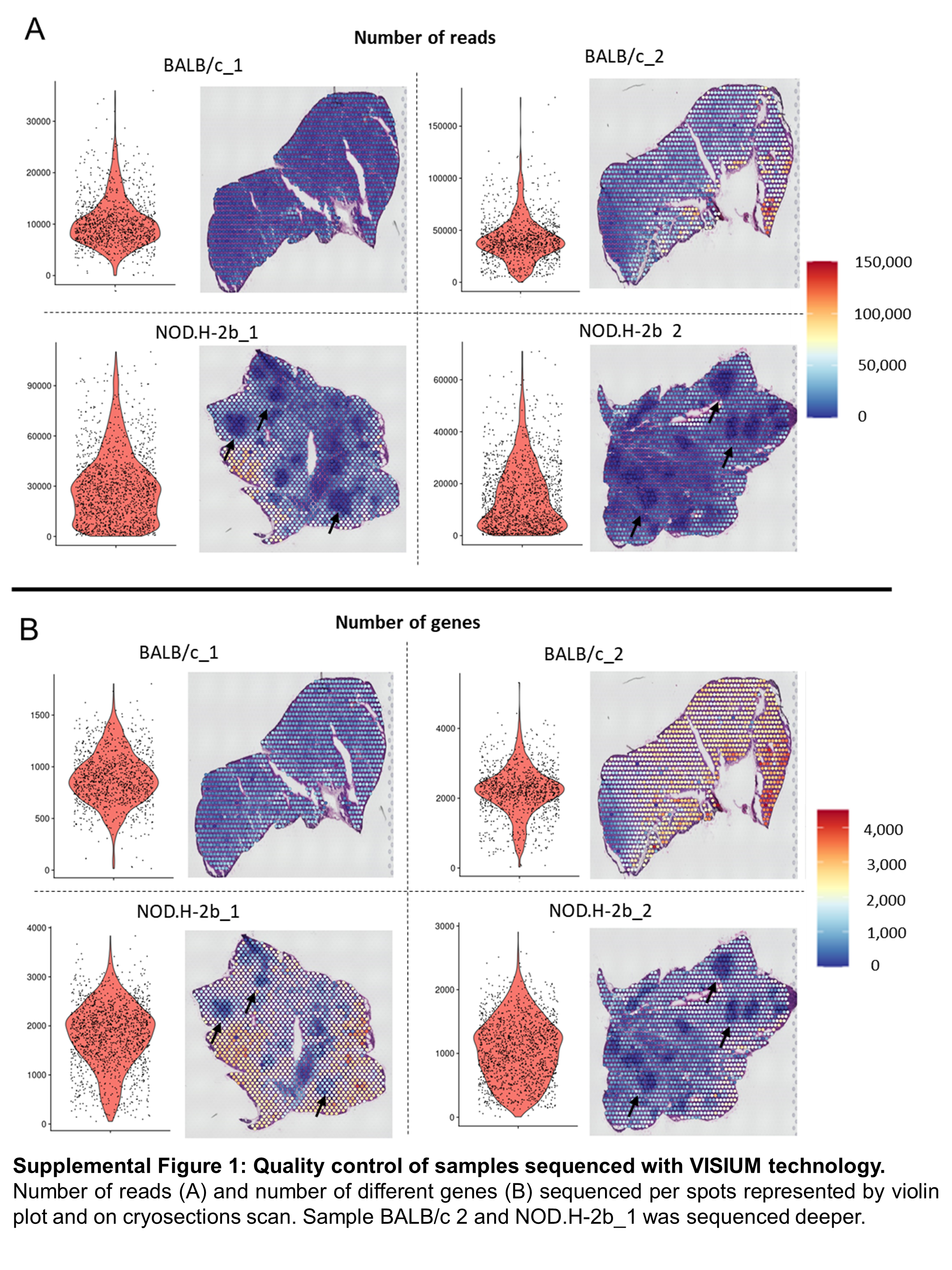

Supplement: Supplementary file 2 [file Image_1.tif]

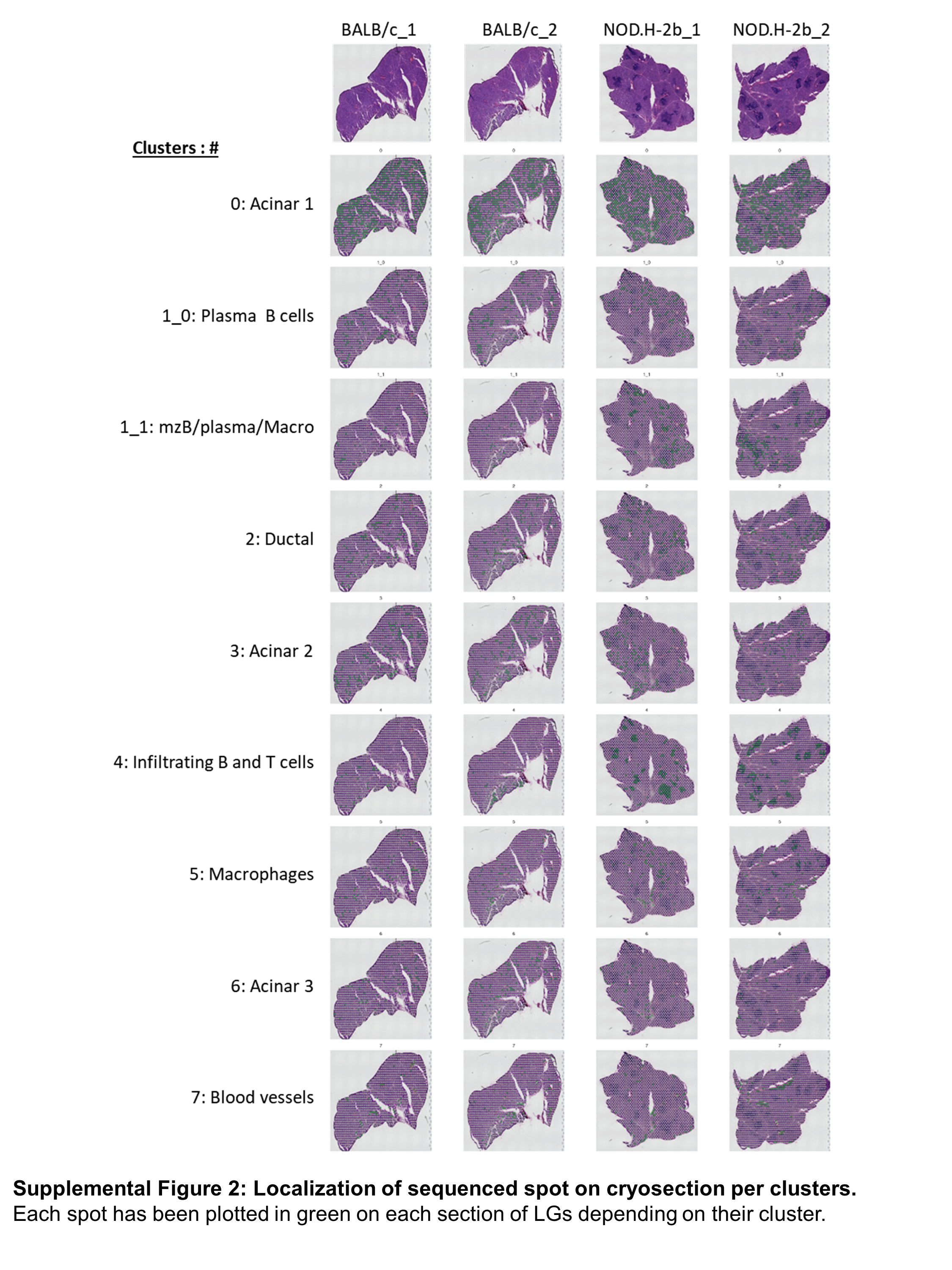

Supplement: Supplementary file 3 [file Image_2.tif]

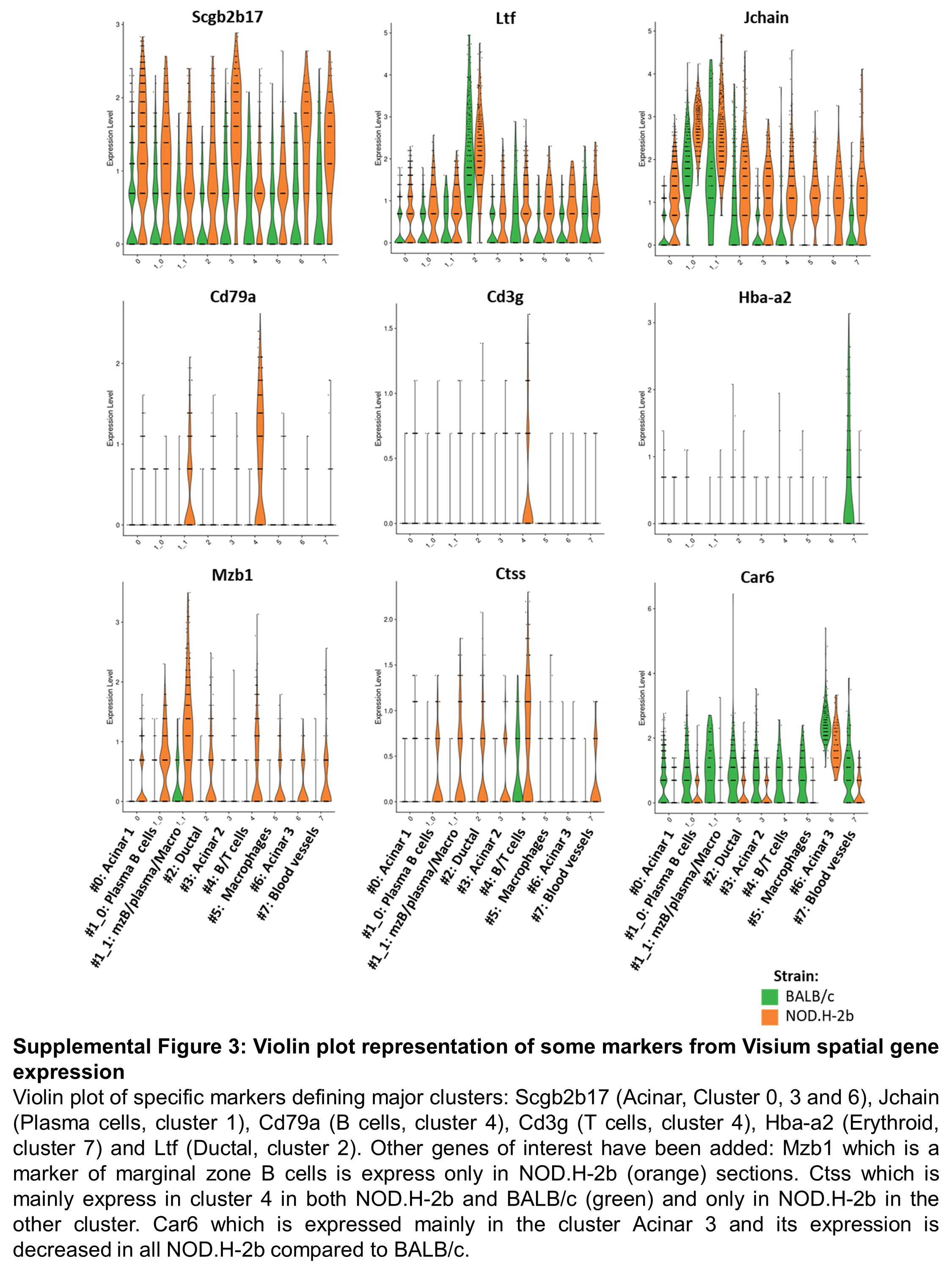

Supplement: Supplementary file 4 [file Image_3.tif]

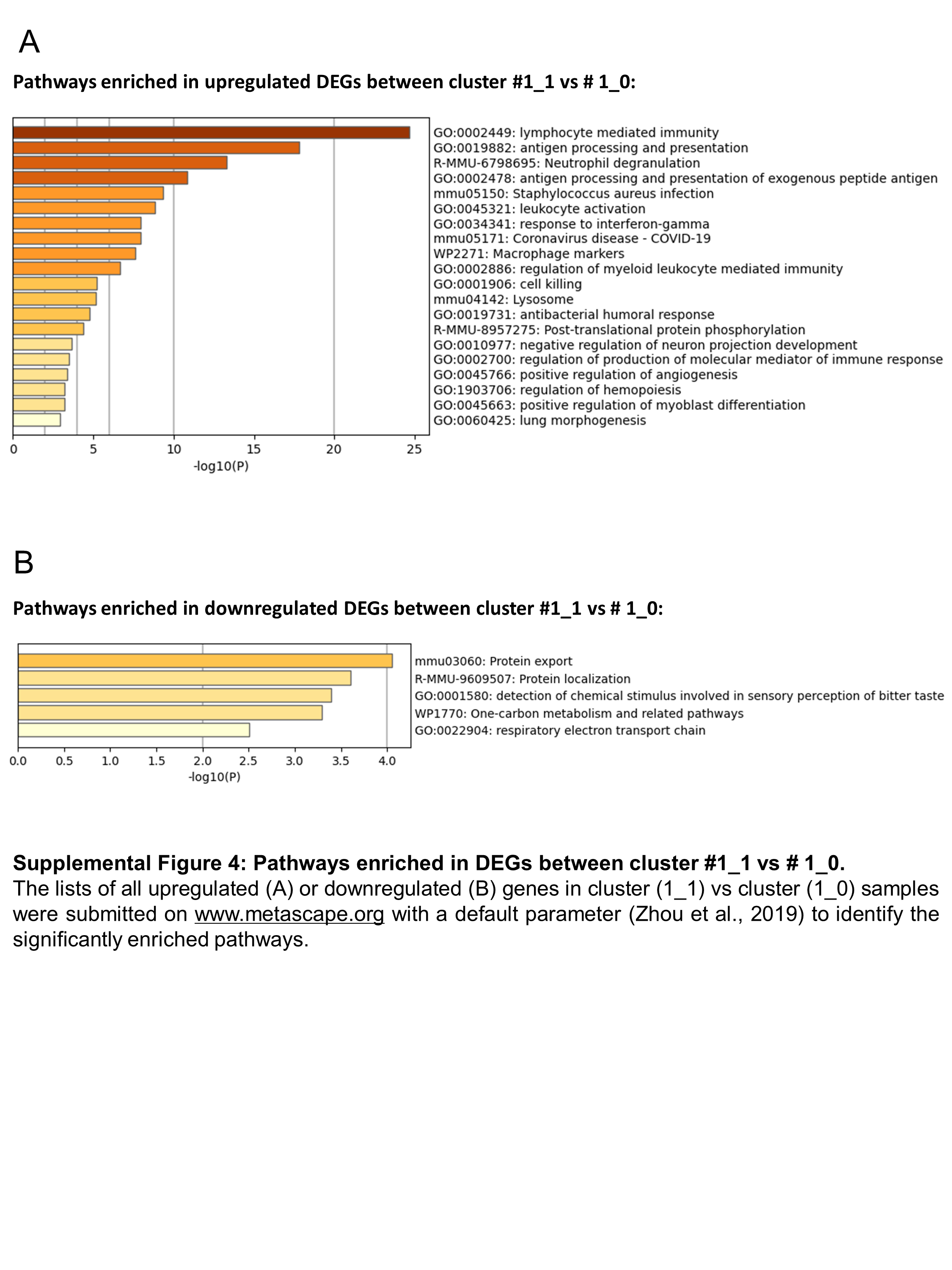

Supplement: Supplementary file 5 [file Image_4.tif]

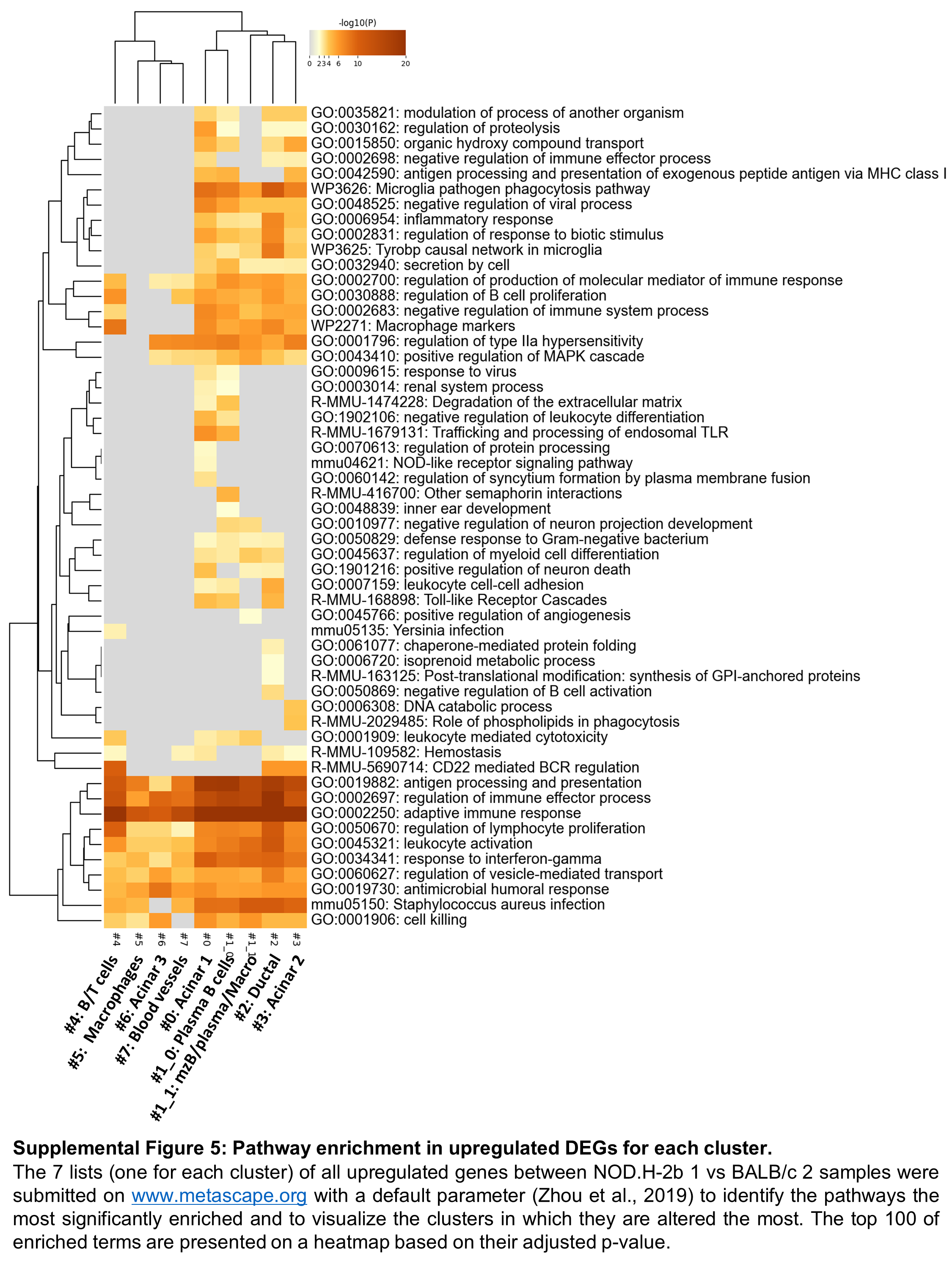

Supplement: Supplementary file 6 [file Image_5.tif]

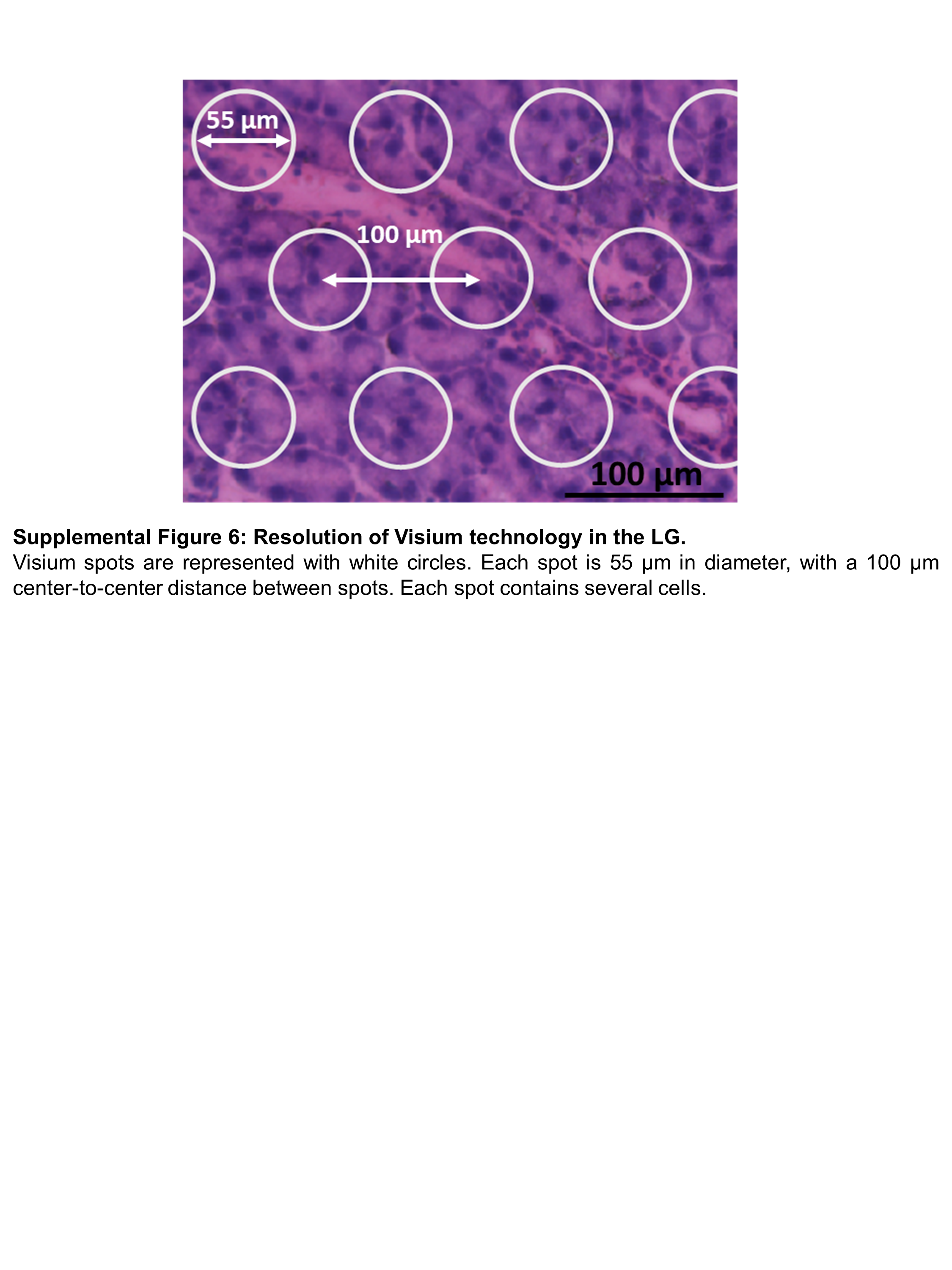

Supplement: Supplementary file 7 [file Image_6.tif]

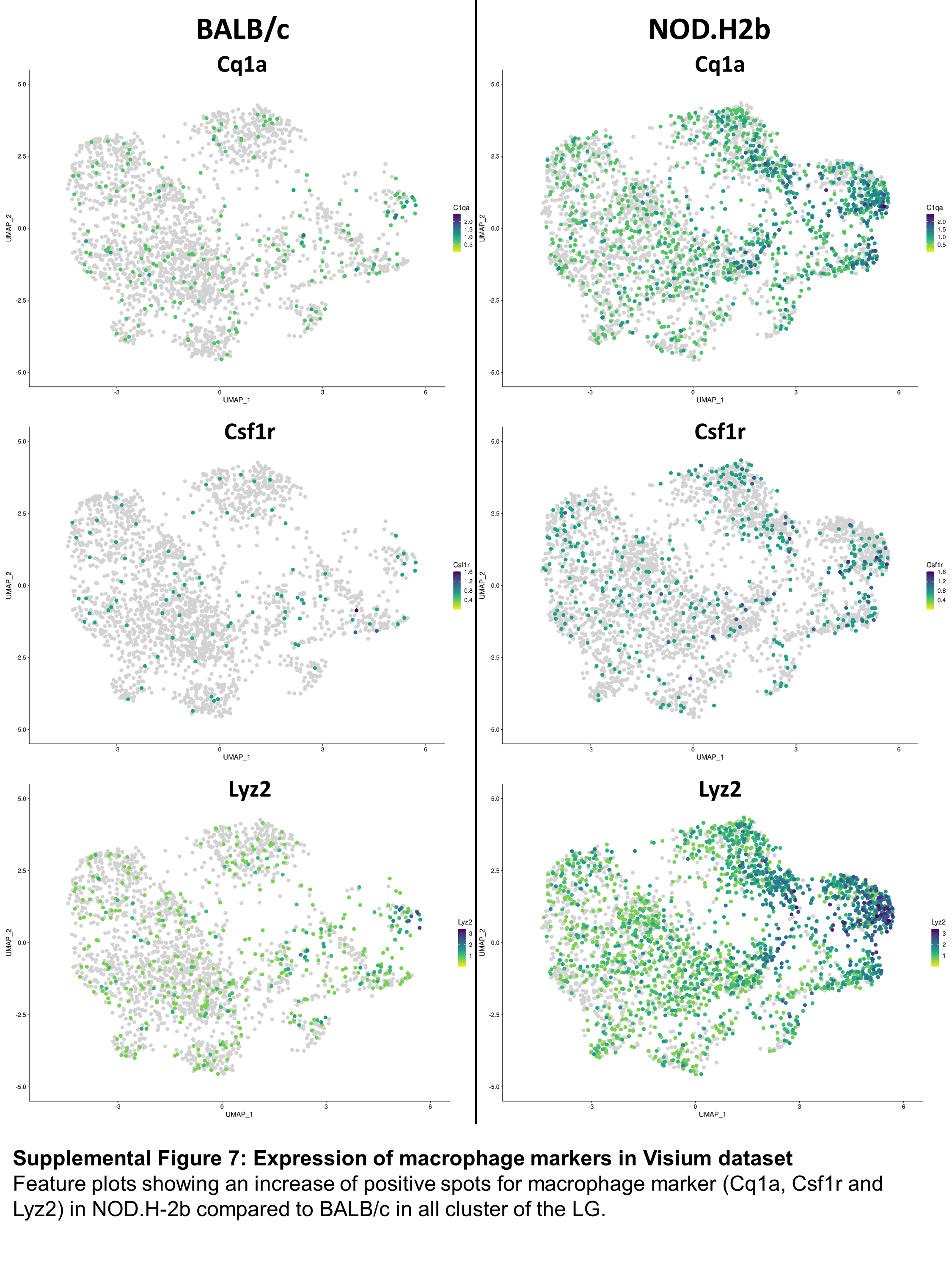

Supplement: Supplementary file 8 [file Image_7.tif]

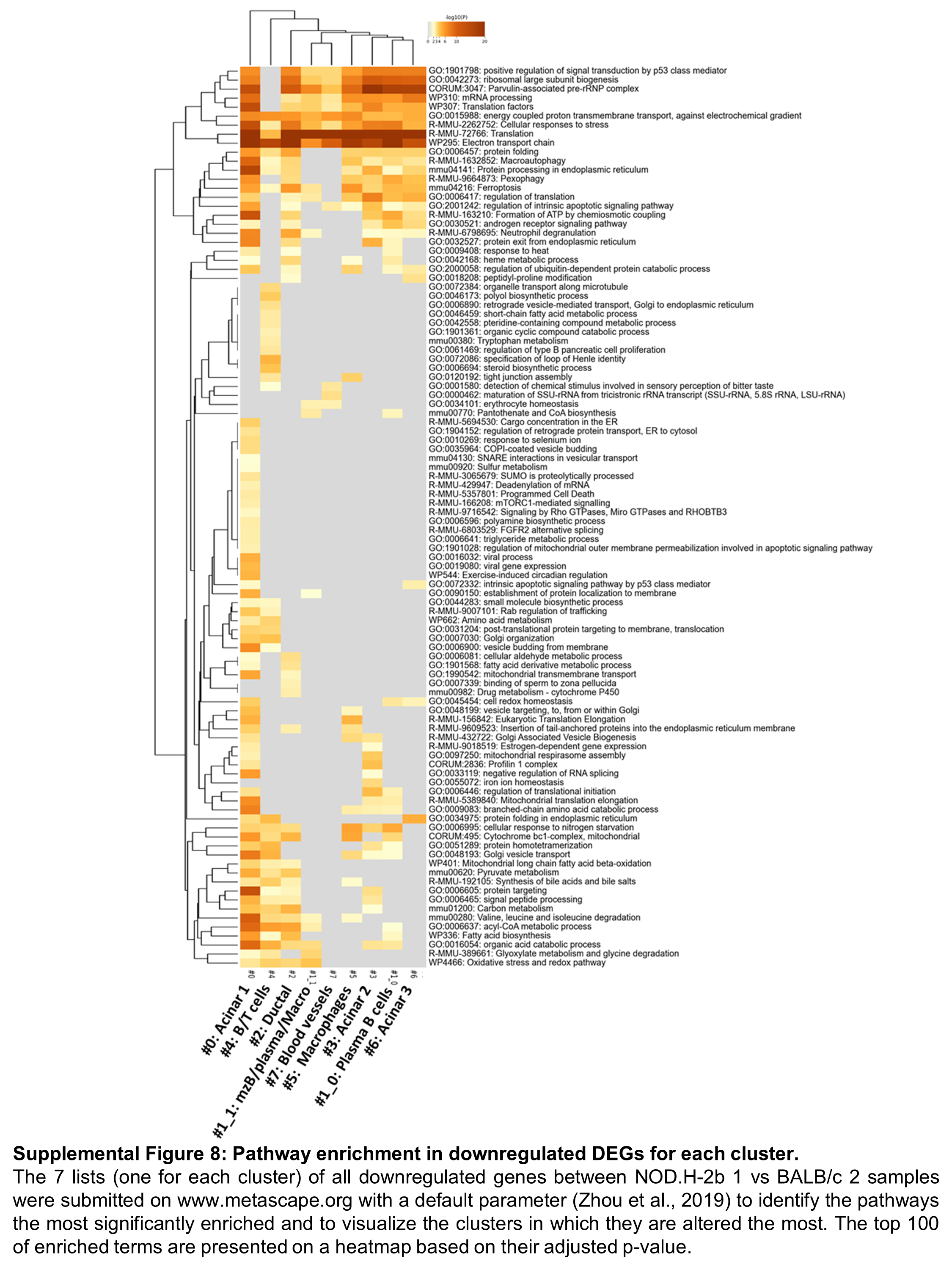

Supplement: Supplementary file 9 [file Image_8.tif]
